# Fabrication of a family of atomically precise silver nanoclusters *via* dual-level kinetic control†

**DOI:** 10.1039/d2sc01016j

**Published:** 2022-04-10

**Authors:** Xiao Wei, Chao Xu, Hao Li, Xi Kang, Manzhou Zhu

**Affiliations:** Department of Chemistry, Centre for Atomic Engineering of Advanced Materials, Key Laboratory of Structure and Functional Regulation of Hybrid Materials of Ministry of Education, Institutes of Physical Science and Information Technology, Anhui Province Key Laboratory of Chemistry for Inorganic/Organic Hybrid Functionalized Materials, Anhui University Hefei Anhui 230601 China kangxi_chem@ahu.edu.cn zmz@ahu.edu.cn

## Abstract

The controllable preparation of metal nanoclusters in high yield is an essential prerequisite for their fundamental research and extensive application. Here a synthetic approach termed “dual-level kinetic control” was developed to fabricate a family of new silver nanoclusters. The introduction of secondary ligands was first exploited to retard the reduction rate and accomplish the first-level kinetic control. And the cooling of the reaction was performed to further slow the reduction down and accomplish the second-level kinetic control. A family of atomically precise silver nanoclusters (including [Ag_25_(SR)_18_]^−^, [Ag_34_(SR)_18_(DPPP)_3_Cl_4_]^2+^, [Ag_36_(SR)_26_S_4_]^2+^, [Ag_37_(SR)_25_Cl_1_]^+^, and [Ag_52_(SR)_28_Cl_4_]^2+^) were controllably prepared and structurally determined. The developed “dual-level kinetic control” hopefully acts as a powerful synthetic tool to manufacture more nanoclusters with unprecedented compositions, structures, and properties.

## Abstract 2

A dual-level kinetic control was exploited to fabricate a family of atomically precise silver nanoclusters.

## Graphical abstract

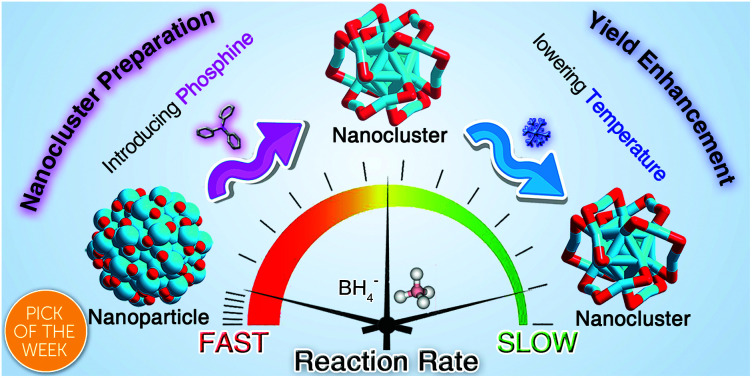

## Introduction

With the recent establishment of modern nanochemistry, capabilities toward dictating the sizes and structures of metal nanoparticles are flourishing.^1–3^ Recent years have witnessed significant advances in the preparation of atomically precise nanoparticles in the quantum size regime, also known as nanoclusters.^4–6^ Metal nanoclusters, bridging between organometallic complexes and plasmonic metal nanoparticles, are a large family of metallo-inorganic-organic hybrid nanomaterials having core@shell structures consisting of internal metal cores and peripheral ligand shells.^7–11^ Owing to their discrete electronic energy levels and the quantum size effect, nanoclusters display molecule-like and structure-dependent chemical/physical properties, rendering them prominent nanomaterials being applied in optics, catalysis, sensing, biochemistry, and so on.^12–24^ Among all research branches, the controllable preparation of metal nanoclusters in high yield (or with high purity) is an essential prerequisite for fundamental research (*e.g.*, structure evolutions and property mechanisms) and extensive applications of these nanomaterials.

In the past few decades, several approaches have been proposed to controllably prepare new clusters with dictated structures and properties, or to efficiently increase the synthetic yields of preexisting clusters, including the “pre-adjusting *in situ* reduction”,^25–27^ the “cluster from clusters”,^28–30^ the “one- or two-phase ligand exchange”,^31–33^ the “anti-galvanic reduction” or “metal exchange”,^34–36^ the “paste-based reaction”,^37,38^ the “cluster-assembled framework”,^39–42^*etc.* Previous experimental and theoretical efforts have demonstrated that the formation of nanocluster entities resulted from both their thermodynamic and kinetic stabilities, which was closely relevant to the reaction environment, especially for the *in situ* synthetic procedure.^43,44^ In 2008, our group developed a facile approach for the preparation of Au_25_(SR)_18_ in a high yield (40%) *via* controlling the reaction kinetics, in vivid contrast against the uncontrolled preparation of Au_25_(SR)_18_ with a low yield (8%).^25^ One challenging question subsequently arises: how can we extend the “kinetic control” to fabricate more nanoclusters, and further improve their synthetic yields by amplifying the “kinetic control”? The in-depth application of such a control would yield more new clusters with novel structures and enhanced properties, significantly assisting the development of this unique class of nanomaterials in terms of both fundamental investigations and practical applications.

Herein, we report the controllable preparation of a family of silver nanoclusters (including [Ag_25_(SR)_18_]^−^, [Ag_34_(SR)_18_(DPPP)_3_Cl_4_]^2+^, [Ag_36_(SR)_26_S_4_]^2+^, [Ag_37_(SR)_25_Cl_1_]^+^, and [Ag_52_(SR)_28_Cl_4_]^2+^) in high yield *via* a “dual-level kinetic control”. Specifically, the direct reduction of Ag_*x*_(S-Adm)_*y*_ complexes produced polydisperse nanoparticles. In contrast, monodispersed nanoclusters could be fabricated *via* “dual-level kinetic control” – (i) the first-level kinetic control: the introduction of phosphine ligands to the reaction retarded the reduction rate, accomplished the kinetic control, and gave rise to different Ag nanoclusters correlating with the phosphine ligand type; (ii) the second-level kinetic control: the cooling of the reaction further reduced the reduction rate, advanced the kinetic control, and remarkably improved the synthetic yields of such Ag clusters. The two-stage braking of the reduction was recorded by tracking photography, and the participation of phosphine ligands in the reduction was verified by mass spectrometry. Together, the dual-level kinetic control enabled the formation of several new silver nanoclusters and further contributed to their high-yield preparation.

## Experimental methods

### Materials

HS-Adm was prepared by the reported procedure.^45^ All the following reagents were purchased from Sigma-Aldrich and used without further purification, including silver nitrate (AgNO_3_, 99% metal basis), triphenylphosphine (PPh_3_, TPP, 99%), bis(diphenylphosphino)methane (Ph_2_P–CH_2_–PPh_2_, DPPM, 98%), 1,2-bis(diphenylphosphino)ethane (Ph_2_P–C_2_H_5_–PPh_2_, DPPE, 98%), 1,3-bis(diphenylphosphino)propane (Ph_2_P–C_3_H_7_–PPh_2_, DPPP, 98%), 1,4-bis(diphenylphosphino)butane (Ph_2_P–C_4_H_9_–PPh_2_, DPPB, 98%), 1,5-bis(diphenylphosphino)pentane (Ph_2_P–C_5_H_11_–PPh_2_, DPPPE, 98%), 1,6-bis(diphenylphosphino)hexane (Ph_2_P–C_6_H_13_–PPh_2_, DPPH, 98%), sodium borohydride (NaBH_4_, 99%), methylene chloride (CH_2_Cl_2_, HPLC grade), methanol (CH_3_OH, HPLC grade), and *n*-hexane (Hex, HPLC grade).

### Reduction of Ag_*x*_(S-Adm)_*y*_ complexes in the absence of phosphine ligands

AgNO_3_ (30 mg) was dissolved in CH_3_OH (1 mL) and CH_2_Cl_2_ (15 mL) by sonication. The solution was vigorously stirred (1200 rpm) with magnetic stirring for 10 min. Then, Adm-SH (0.1 g) was added and the reaction was vigorously stirred (1200 rpm) for another 30 min. After that, NaBH_4_ (1 mL) aqueous solution (20 mg mL^−1^) was added quickly to the above reaction. The reaction was allowed to proceed for 12 h under a N_2_ atmosphere. Then, the precipitate was removed and the supernatant was analyzed by STEM.

### Preparation of a family of silver nanoclusters in the presence of phosphine ligands

AgNO_3_ (30 mg) was dissolved in CH_3_OH (1 mL) and CH_2_Cl_2_ (15 mL) by sonication. The solution was vigorously stirred (1200 rpm) with magnetic stirring for 10 min. Then, Adm-SH (0.1 g) and the phosphine ligand (0.1 g) were added together and the reaction was vigorously stirred (1200 rpm) for another 30 min at room temperature or in an ice bath. After that, NaBH_4_ (1 mL) aqueous solution (20 mg mL^−1^) was added quickly to the above reaction. The reaction was allowed to proceed for 12 h under a N_2_ atmosphere. After that, the aqueous layer was removed, and the mixture in the organic phase was rotavaporated under vacuum. Then, approximately 15 × 3 mL of CH_3_OH was used to wash the synthesized nanoclusters. The precipitate was then dissolved in CH_2_Cl_2_ for crystallization and characterization. Different silver nanoclusters were synthesized correlating with the phosphine ligand type, including [Ag_52_(S-Adm)_28_Cl_4_]^2+^ by using TPP or DPPPE, [Ag_36_(S-Adm)_26_S_4_]^2+^ by using DPPM, [Ag_25_(S-Adm)_18_][Ag_1_(DPPE)_2_] by using DPPE, [Ag_34_(S-Adm)_18_(DPPP)_3_Cl_4_]^2+^ by using DPPP, [Ag_37_(S-Adm)_25_Cl_1_]^+^ by using DPPB, and [Ag_25_(S-Adm)_18_][Ag_3_(S-Adm)_2_(DPPH)_2_] by using DPPH. The synthetic yields were calculated on Ag basis by analyzing the qualities of Ag nanocluster crystals and the AgNO_3_ salts. Such synthetic yields are presented in Fig. 2B.

**Fig. 1:**
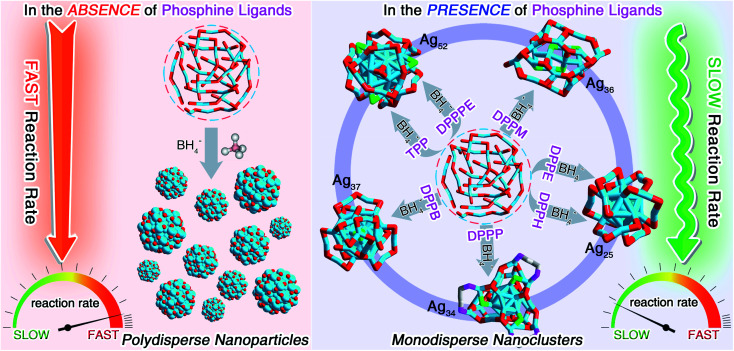
Schematic illustration of the first-level kinetic control *via* introducing phosphine ligands. Controllable preparation of a family of silver nanoclusters by retarding the reaction rate through the introduction of phosphine ligands.

**Fig. 2:**
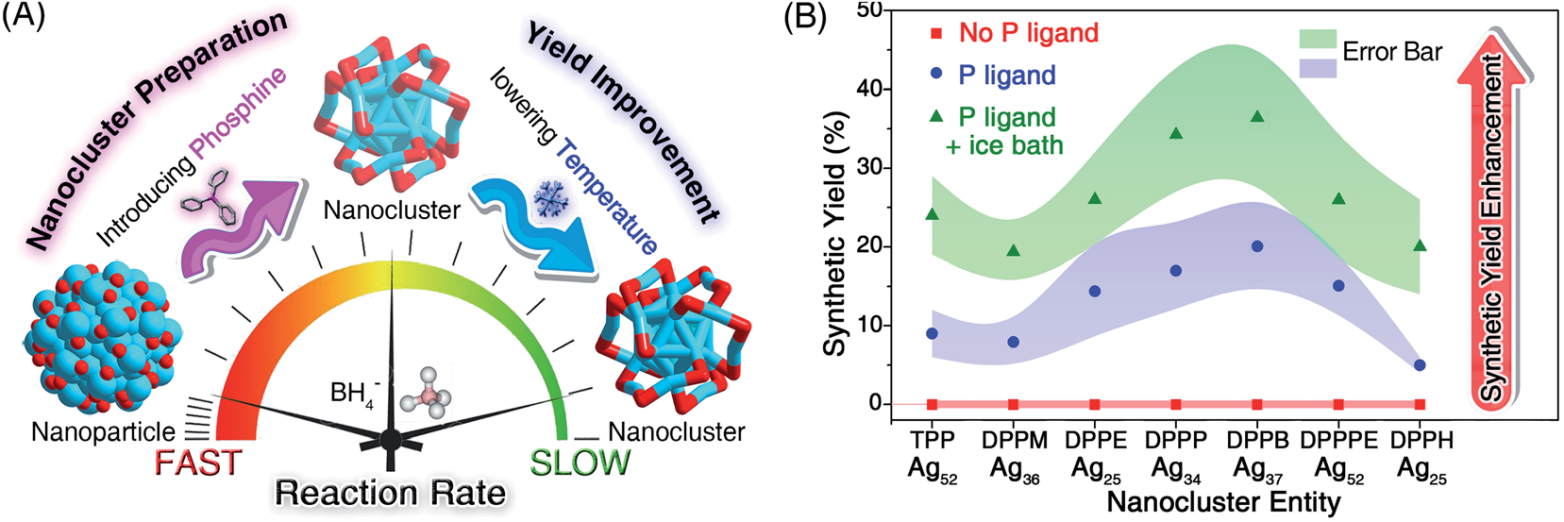
The dual-level kinetic control for the preparation of silver nanoclusters. (A) Illustration of the controllable preparation of Ag nanoclusters by retarding the reaction rate through introducing phosphine ligands (*i.e.*, nanocluster preparation *via* the first-level kinetic control) and lowering the reaction temperature (*i.e.*, yield improvement *via* the second-level kinetic control). (B) Synthetic yields of Ag nanoclusters under different conditions: in the absence of phosphine ligands at room temperature (red region), in the presence of phosphine ligands at room temperature (blue region), and in the presence of phosphine ligands in an ice bath (green region).

### Crystallization of the obtained silver nanoclusters

Single crystals of the obtained silver nanoclusters were grown at room temperature in CH_2_Cl_2_/Hex. Of note, for accelerating the crystallization processes of Ag_36_(S-Adm)_26_S_4_ and Ag_34_(S-Adm)_18_(DPPP)_3_Cl_4_ and improving their crystal quality, the original counterion was replaced by SbF_6_^−^. After 7 days, black crystals of these clusters were collected and their structures were determined by X-ray crystallography.

### X-ray crystallography

The data collection for single-crystal X-ray diffraction (SC-XRD) of all nanocluster crystal samples was carried out on a Stoe Stadivari diffractometer under a nitrogen flow, using graphite-monochromatized Cu Kα radiation (*λ* = 1.54186 Å). Data reductions and absorption corrections were performed using the SAINT and SADABS programs, respectively. The structure was solved by direct methods and refined with full-matrix least squares on *F*^2^ using the SHELXTL software package. All non-hydrogen atoms were refined anisotropically, and all the hydrogen atoms were set in geometrically calculated positions and refined isotropically using a riding model. All crystal structures were treated with PLATON SQUEEZE, and the diffuse electron densities from these residual solvent molecules were removed. The CCDC number of the Ag_52_(S-Adm)_28_Cl_4_ nanocluster (prepared in the presence of TPP) is 2094270. The CCDC number of Ag_36_(S-Adm)_28_S_4_ is 2094271. The CCDC number of [Ag_25_(S-Adm)_18_][Ag_1_(DPPE)_2_] is 2094272. The CCDC number of Ag_34_(S-Adm)_18_(DPPP)_3_Cl_4_ is 2094273. The CCDC number of Ag_37_(S-Adm)_25_Cl_1_ is 2094275. The CCDC number of the Ag_52_(S-Adm)_28_Cl_4_ nanocluster (prepared in the presence of DPPPE) is 2094276. The CCDC number of [Ag_25_(S-Adm)_18_][Ag_3_(S-Adm)_2_(DPPH)_2_] is 2094482.

### Measurements

All UV-vis absorption spectra of the nanoclusters dissolved in CH_2_Cl_2_ were recorded using an Agilent 8453 diode array spectrometer.

The dynamic light scattering (DLS) of each metal complex sample was recorded using a Malvern Zetasizer Nano ZS instrument.

Electrospray ionization mass spectrometry (ESI-MS) measurements were performed by using a Waters XEVO G2-XS QTof mass spectrometer. The sample was directly infused into the chamber at 5 μL min^−1^. For preparing the ESI samples, nanoclusters were dissolved in CH_2_Cl_2_ (1 mg mL^−1^) and diluted (v/v = 1 : 1) with CH_3_OH.

Thermogravimetric analysis (TGA) was carried out on a thermogravimetric analyzer (DTG-60H, Shimadzu Instruments, Inc.) with 10 mg of the sample in a SiO_2_ pan at a heating rate of 10 K min^−1^ from room temperature to 1073 K.

The high angle annular dark field scanning transmission electron microscopy (HAADF-STEM) technique was performed by using a FEI Themis Z microscope. The electron beam energy was 200 kV. The HAADF-STEM image was obtained using Thermo Scientific Velox software using 1024 × 1024 pixels and the dwell time was set to 10 μs.

## Results and discussion

### The first-level kinetic control

We first directly reduced the Ag_*x*_(S-Adm)_*y*_ complexes using NaBH_4_ for the sake of preparing new silver nanoclusters (see Methods for more details). However, the reaction was ultra-violent after the introduction of the reductant, and the reaction turned black within three seconds, accompanied by the formation of massive precipitation (Fig. S1†). Previous studies have demonstrated the time-dependent size aggregation of nanoclusters by the synthetic procedure,^46–48^ while the resultant products of this reaction remained polydisperse nanoclusters (or nanoparticles) in the range from 1 to 10 nm, even after 12 hours of the reaction (Fig. S2A†). The TGA result demonstrated that the Ag-to-SAdm ratio of these nanoparticles was 16.67% (Fig. S2B†). Through observation of the reaction phenomena, we suspected that the unsuccessful attempt at the monodispersed nanocluster preparation resulted from the over-quick reduction that generated excessively heterogeneous metallic nuclei. Besides, such an ultrafast reduction may also yield large-sized metallic nuclei, which were responsible for the formation of massive precipitation in the reaction. Accordingly, the core issue herein for the preparation of monodispersed nanoclusters lies in retarding the reduction rate, or in other words the kinetic control.

For fulfilling the kinetic control of the reaction, we introduced phosphine ligands to the reaction for two reasons – (i) the sizes of Ag–(S-Adm)–PR complexes before the reduction were prone to be more concentrated, while those of Ag–(S-Adm) complexes were much more dispersed (Fig. S3†). The more size-focused Ag–(S-Adm)–PR complexes were more accessible to be reduced uniformly, and thus produced monodispersed nanoclusters. Besides, the sizes of such Ag–(S-Adm)–PR complexes were, generally, in inverse proportion to the sizes of phosphine ligands. (ii) The coexistence of different ligands in metal complex precursors (*i.e.*, thiol and phosphine co-protected complexes, as shown in Fig. S4 and S5†) might prevent the rapid formation of large-sized metallic nuclei due to their complex stabilizing patterns, holding the potential to retard the reduction rate and accomplish the kinetic control.^49^ Experimentally, the reduction rate was remarkably reduced in the presence of phosphine ligands (Fig. S6–S12†). For instance, the reaction solution turned black after three minutes when TPP or DPPM ligands were involved (Fig. S6 and S7†), which was in stark contrast to the three-second mutation in the absence of phosphine ligands. Such a kinetic control resulted in the mild reaction environment and gave rise to different silver nanoclusters correlating with the phosphine ligand type, including [Ag_25_(SR)_18_]^−^, [Ag_34_(SR)_18_(DPPP)_3_Cl_4_]^2+^, [Ag_36_(SR)_26_S_4_]^2+^, [Ag_37_(SR)_25_Cl_1_]^+^, and [Ag_52_(SR)_28_Cl_4_]^2+^. The formation of different Ag nanoclusters in the presence of different phosphine ligands resulted from the ligand selection effect, corresponding to the “survival of the fittest” in the thermodynamically selective synthesis. Collectively, the kinetic control was accomplished *via* introducing phosphine ligands to the reaction (termed “first-level kinetic control” in this work), resulting in the control of nanocluster sizes and the formation of a family of monodispersed silver nanoclusters (Fig. 1).

### The second-level kinetic control

Although the first-level kinetic control (*i.e.*, introducing phosphine ligands) resulted in monodispersed nanoclusters rather than polydisperse nanoparticles, the synthetic yields of these nanoclusters were relatively low (∼10% yield for each Ag cluster; see Fig. 2). Based on the above understanding that the slowing down of the reduction rate induced the formation of nanoclusters, we perceive a good opportunity to further advance the kinetic control (or to implement the second-level kinetic control) for increasing the synthetic yield of these Ag nanoclusters – lowering the temperature of the reaction *via* an ice bath.

As shown in Fig. S6–S12,† cooling the reaction could remarkably slow down the reaction rate. For example, the time required for turning the solution black for the preparation of Ag_25_(S-Adm)_18_ even doubled with the ice bath (Fig. S12†). After analyzing the synthetic yield of each case for the nanocluster preparation, we concluded that the proposed second-level kinetic control (*i.e.*, cooling the reaction) was capable of increasing the yield of each silver nanocluster (Fig. 2B). For example, the synthetic yields of Ag_52_ in the presence of TPP or DPPPE were enhanced from ∼10% to ∼25% or from ∼15% to ∼27%, respectively. The synthetic yields of other Ag nanoclusters also exhibited different degrees of enhancement with the second-level kinetic control (Fig. 2B). In this context, a combination of dual-level kinetic controls was exploited to fabricate a family of monodispersed silver nanoclusters and further improve their synthetic yields (Fig. 2A).

### Structural anatomy of the [Ag_52_(S-Adm)_28_Cl_4_]^2+^ nanocluster

**Fig. 3:**
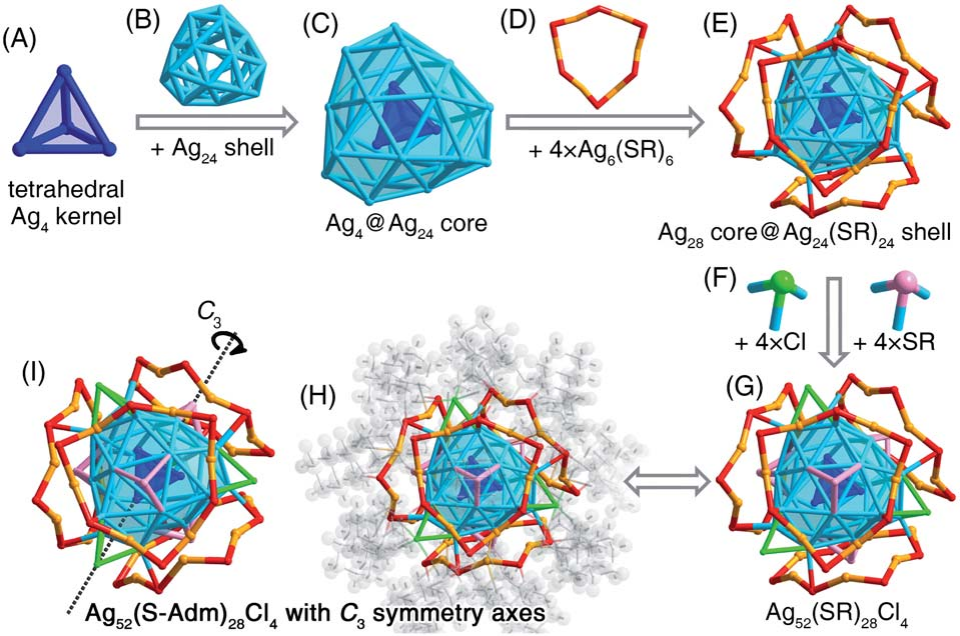
Structural anatomy of [Ag_52_(S-Adm)_28_Cl_4_]^2+^. (A) The tetrahedral Ag_4_ kernel + (B) the Ag_24_ shell = (C) the Ag_28_ core. (D) The Ag_6_(SR)_6_ ring-like motif. (E) The Ag_52_(SR)_24_ structure. (F) Surface Cl or SR bridges. (G) The Ag_52_(SR)_28_Cl_4_ framework. (H) and (I) The overall structure of [Ag_52_(S-Adm)_28_Cl_4_]^2+^ exhibits four *C*_3_ symmetry axes. Color legends: blue/light blue/orange sphere, Ag; red/pink sphere, S; green sphere, Cl; grey sphere, C; light grey sphere, H.

The presence of monodentate TPP or bidentate DPPPE ligands in the reaction gave rise to the formation of the [Ag_52_(S-Adm)_28_Cl_4_]^2+^ nanocluster (Fig. 2B and S13†). The [Ag_52_(S-Adm)_28_Cl_4_]^2+^ cluster entities are crystallized in an orthorhombic crystal system with a *Pccn* space group. Structurally, [Ag_52_(S-Adm)_28_Cl_4_]^2+^ contains a tetrahedral Ag_4_ kernel that is enwrapped by an Ag_24_ shell (Fig. 3A–C). Such a two-shell Ag_4_@Ag_24_ configuration has been previously observed in Ag_28_Cu_12_(SPhCl_2_)_24_ and Cd_12_Ag_32_(SePh)_36_ nanoclusters.^50–52^ Then, this Ag_28_ core is encircled by four same Ag_6_(S-Adm)_6_ ring-like surface motif structures to form an Ag_28_(core)@Ag_24_(S-Adm)_24_(shell) structure (Fig. 3D and E). Of note, the Ag_24_(S-Adm)_24_ shell structure in Ag_52_(S-Adm)_28_Cl_4_, composed of hexameric Ag_6_(S-Adm)_6_ surface motifs, is relatively loose compared to 4 × [Cu_3_(SPhCl_2_)_6_] or 4 × [Cd_3_Ag_1_(SePh)_9_] surface structures in Ag_28_Cu_12_(SPhCl_2_)_24_ and Cd_12_Ag_32_(SePh)_36_ nanoclusters, respectively. In this context, three Cl and three S-Adm ligands, acting as bridges, fill up surface spaces (*i.e.*, the exposed Ag_3_ triangles on the Ag_4_@Ag_24_ core) on the Ag_28_@Ag_24_(S-Adm)_24_ structure to make up the final [Ag_52_(S-Adm)_28_Cl_4_]^2+^ framework (Fig. 3F and G). The Cl ligands are proposed to originate from the CH_2_Cl_2_ solvent, which has been discovered in previously determined nanoclusters,^53,54^ and Ag_34_ and Ag_37_ nanoclusters in this work. The overall structure of [Ag_52_(S-Adm)_28_Cl_4_]^2+^ is highly symmetrical with four *C*_3_ symmetry axes, and each symmetry axis passes through the center of the innermost Ag_4_ kernel and polar S/Cl atoms at contrapositions (Fig. 3H and I).

### Structural anatomy of the [Ag_36_(S-Adm)_26_S_4_]^2+^ nanocluster

**Fig. 4:**
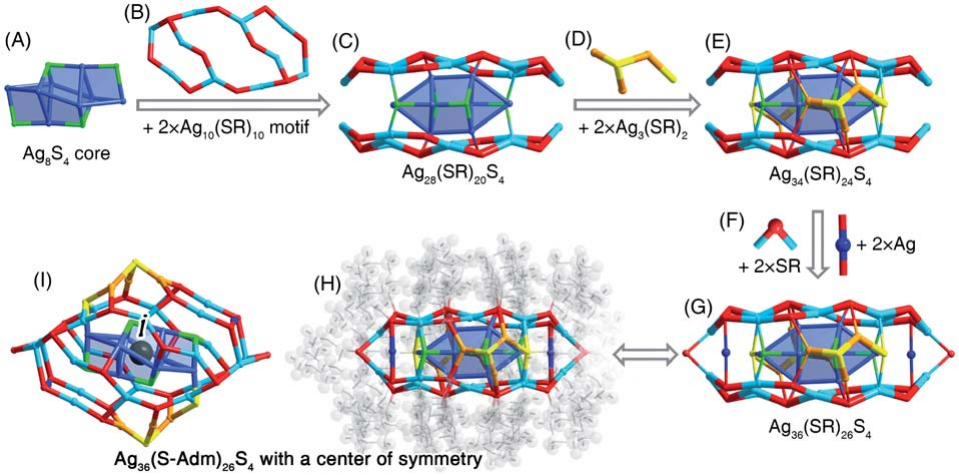
Structural anatomy of [Ag_36_(S-Adm)_26_S_4_]^2+^. (A) The Ag_6_S_4_ core + (B) two Ag_10_(SR)_10_ surface motifs = (C) the Ag_28_(SR)_20_S_4_ structure. (D) The Ag_3_(SR)_2_ side motif structures. (E) The Ag_34_(SR)_24_S_4_ structure. (F) The bridging SR and Ag units. (G) The Ag_36_(SR)_26_S_4_ framework. (H) and (I) The overall structure of [Ag_36_(S-Adm)_26_S_4_]^2+^ exhibits a center of symmetry. Color legends: blue/light blue/orange sphere, Ag; red/yellow sphere, S; grey sphere, C; light grey sphere, H.

The [Ag_36_(S-Adm)_26_S_4_]^2+^ nanocluster was prepared when DPPM was introduced to the reaction (Fig. 2B and S14†). The [Ag_36_(S-Adm)_26_S_4_]^2+^ cluster entities are crystallized in a monoclinic crystal system with a *P*2_1_/*n* space group. The structure of Ag_36_(S-Adm)_26_S_4_ comprises an anti-*z*-shaped Ag_8_S_4_ core that is covered by two Ag_10_(S-Adm)_10_ motif structures *via* both Ag(core)–S(motif) and Ag(motif)–S(core) interactions, making up an Ag_28_(S-Adm)_20_S_4_ structure (Fig. 4A–C). The sulfurs without carbon tails should stem from the thiol (*i.e.*, S-Adm) in the formation of the nanoclusters, which has been detected in previously reported nanoclusters, such as Au_38_S_2_(SR)_20_, Au_60_S_6_(SR)_36_, Ag_46_S_7_(SR)_24_, *etc.*^55–57^ Then, the side of the Ag_28_(S-Adm)_20_S_4_ structure is stabilized by two Ag_3_(SR)_2_ units (Fig. 4D and E), and the two symmetrical Ag_10_(S-Adm)_10_ motifs are held together *via* two SR and two Ag bridges, constituting the Ag_36_(S-Adm)_26_S_4_ framework with a flattened configuration (Fig. 4F and G). No symmetrical element except for a center of symmetry is observed for the overall structure of the [Ag_36_(S-Adm)_26_S_4_]^2+^ nanocluster, which is located at the center of the Ag_6_S_4_ core (Fig. 4H and I).

### Structural anatomy of the [Ag_25_(S-Adm)_18_]^−^ nanocluster

**Fig. 5:**
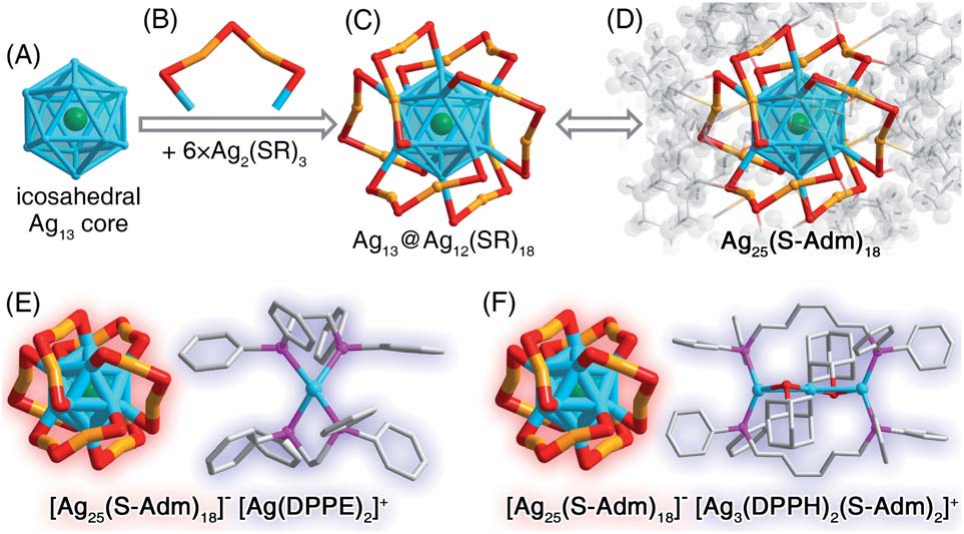
Structural anatomy of [Ag_25_(S-Adm)_18_]^−^. (A) The icosahedral Ag_13_ kernel + (B) six Ag_2_(SR)_3_ dimeric motifs = (C) the Ag_25_(SR)_18_ framework. (D) The overall structure of Ag_25_(S-Adm)_18_. (E) The overall structure of [Ag_25_(SR)_18_]^−^[Ag_1_(DPPE)_2_]^+^. (F) The overall structure of [Ag_25_(SR)_18_]^−^[Ag_3_(DPPH)_2_(SR)_2_]^+^. Color legends: green/light blue/orange sphere, Ag; red sphere, S; magenta sphere, P; grey sphere, C; light grey sphere, H.

The presence of DPPE or DPPH ligands yielded [Ag_25_(S-Adm)_18_]^−^ nanoclusters (Fig. 2B, S15 and S16†), while the counterions were different – [Ag_1_(DPPE)_2_]^+^ for the DPPE-associate [Ag_25_(S-Adm)_18_]^−^, or [Ag_3_(DPPH)_2_(S-Adm)_2_]^+^ for the DPPH-associate [Ag_25_(S-Adm)_18_]^−^ (Fig. 5, S15 and S16†). Structurally, the [Ag_1_(DPPE)_2_]^+^ cation follows a configuration of DPPE(side)–Ag(center)–DPPE(side) (Fig. 5E); by comparison, the [Ag_3_(DPPH)_2_(S-Adm)_2_]^+^ cation contains a linear Ag_3_ structure wherein the two side Ag atoms are bonded with DPPH, and the side and central Ag atoms are anchored by S-Adm (Fig. 5F). The [Ag_25_(S-Adm)_18_][Ag_1_(DPPE)_2_] cluster entities are crystallized in a triclinic crystal system with a *P*1̄ space group, whereas the [Ag_25_(S-Adm)_18_][Ag_3_(DPPH)_2_(S-Adm)_2_] cluster entities are crystallized in a monoclinic crystal system with a *P*2_1_/*c* space group.

The [Ag_25_(S-Adm)_18_]^−^ nanocluster is composed of an icosahedral Ag_13_ kernel and six peripheral Ag_2_(S-Adm)_3_ dimeric motifs (Fig. 5A–D), which is reminiscent of the previously reported [Ag_25_(S-PhMe_2_)_18_]^−^ nanocluster with the same metal–ligand composition and configuration, but with different thiol ligand types.^58^ Here, the corresponding bond lengths in different Ag_25_(SR)_18_ nanoclusters were compared to figure out the ligand effect on the geometric structure of this nanocluster. As depicted in Table S8,† both the kernel Ag–icosahedral Ag and the icosahedral Ag–icosahedral Ag bonds in [Ag_25_(S-Adm)_18_]^−^ are much longer than those in [Ag_25_(S-PhMe_2_)_18_]^−^, while both icosahedral Ag–motif S and motif Ag–motif S bonds in [Ag_25_(S-Adm)_18_]^−^ are much shorter. Accordingly, compared with [Ag_25_(S-PhMe_2_)_18_]^−^, [Ag_25_(S-Adm)_18_]^−^ displays a more expansive kernel structure while a tighter kernel–surface interaction; in other words, the bulkier S-Adm ligand endows the Ag_25_(SR)_18_ nanocluster with a loose inside@tight outside intracluster environment.

### Structural anatomy of the [Ag_34_(S-Adm)_18_(DPPP)_3_Cl_4_]^2+^ nanocluster

The presence of DPPP in the reaction gave rise to the formation of the [Ag_34_(S-Adm)_18_(DPPP)_3_Cl_4_]^2+^ nanocluster (Fig. 2B and S17†). The [Ag_34_(S-Adm)_18_(DPPP)_3_Cl_4_]^2+^ cluster entities are crystallized in a trigonal crystal system with a *R*3̄ space group. Of note, as for all silver nanoclusters in this work, only the [Ag_34_(S-Adm)_18_(DPPP)_3_Cl_4_]^2+^ nanocluster contains a phosphine ligand within the structure. In contrast, in other Ag nanoclusters, the phosphine ligands can not only slow down the reduction rate, but also act as a “dam” to temporarily store Ag and then release it to generate the nanoclusters.^49^

**Fig. 6:**
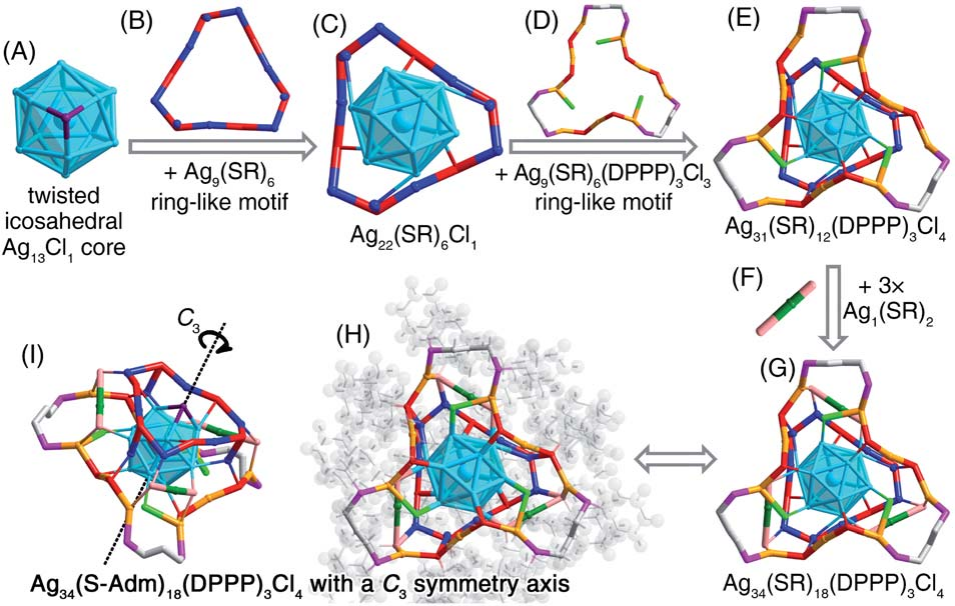
Structural anatomy of [Ag_34_(S-Adm)_18_(DPPP)_3_Cl_4_]^2+^. (A) The twisted icosahedral Ag_13_Cl_1_ core + (B) the Ag_9_(SR)_6_ ring-like motif = (C) The Ag_22_(SR)_6_Cl_1_ structure. (D) The Ag_9_(SR)_6_(DPPP)_3_Cl_3_ ring-like motif. (E) The Ag_31_(SR)_12_(DPPP)_3_Cl_4_ structure. (F) Three bridging Ag_1_(SR)_2_ units. (G) The Ag_34_(SR)_18_(DPPP)_3_Cl_4_ framework. (H) and (I) The overall structure of [Ag_34_(S-Adm)_18_(DPPP)_3_Cl_4_]^2+^ exhibits a *C*_3_ symmetry axis. Color legends: light blue/blue/orange/green sphere, Ag; red/pink sphere, S; purple sphere, Cl; magenta sphere, P; grey sphere, C; light grey sphere, H.

Structurally, the [Ag_34_(S-Adm)_18_(DPPP)_3_Cl_4_]^2+^ nanocluster contains a twisted icosahedral Ag_13_Cl_1_ core (Fig. 6A). The three Ag atoms connecting the Cl ligand are unbound among each other, rendering the Ag_13_ icosahedron twisted. Then, two types of ring-like motif structures, Ag_9_(S-Adm)_6_ and Ag_9_(S-Adm)_6_(DPPP)_3_Cl_3_, enwrap the Ag_13_Cl_1_ core from opposite faces to constitute an Ag_31_(S-Adm)_12_(DPPP)_3_Cl_4_ structure (Fig. 6B–E). Three monomeric Ag_1_(S-Adm)_2_ bridges are further introduced to fix the two ring-like motifs and fully protect the Ag_13_Cl_1_ core, making up the final Ag_34_(S-Adm)_18_(DPPP)_3_Cl_4_ framework (Fig. 6F and G). The overall configuration of [Ag_34_(S-Adm)_18_(DPPP)_3_Cl_4_]^2+^ is triple axisymmetric, and the *C*_3_ symmetry axis crosses through the Cl atom and the innermost Ag atom in the Ag_13_Cl_1_ core (Fig. 6H and I).

### Structural anatomy of the [Ag_37_(S-Adm)_25_Cl_1_]^+^ nanocluster

**Fig. 7:**
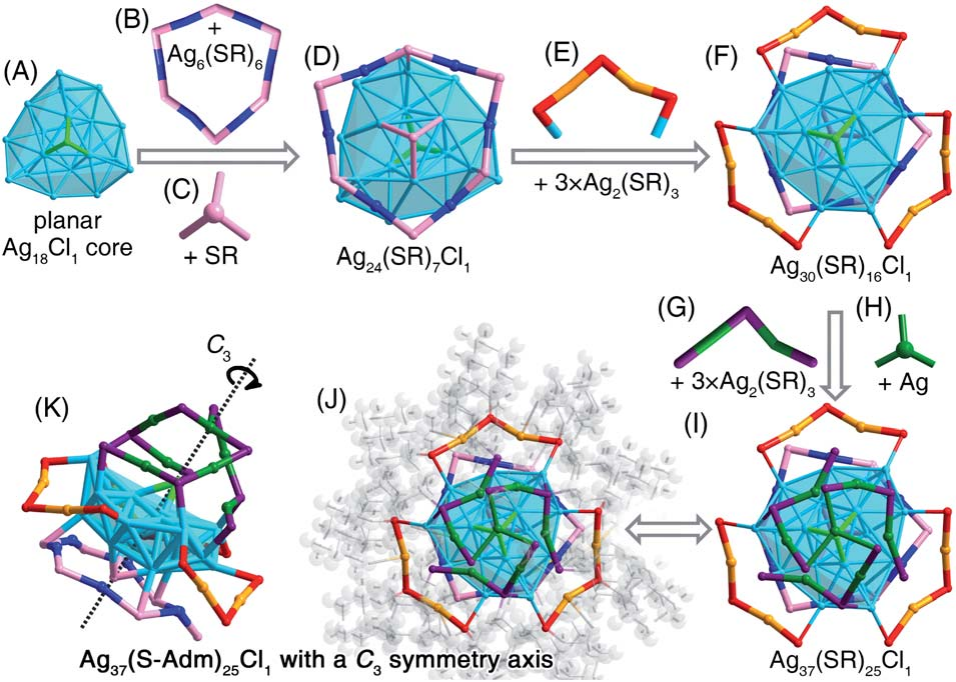
Structural anatomy of Ag_37_(S-Adm)_25_Cl_1_. (A) The planar Ag_18_Cl_1_ core + (B) the Ag_6_(SR)_6_ ring-like motif + (C) the capping SR = (D) The Ag_24_(SR)_7_Cl_1_ structure. (E) Three Ag_2_(SR)_3_ dimeric motifs. (F) The Ag_30_(SR)_16_Cl_1_ structure. (G) Three Ag_2_(SR)_3_ dimeric motifs. (H) The bridging Ag. (I) The Ag_37_(SR)_25_Cl_1_ framework. (J) and (K) The overall structure of Ag_37_(S-Adm)_25_Cl_1_ exhibits a *C*_3_ symmetry axis. Color legends: light blue/blue/orange/green sphere, Ag; pink/orange/purple sphere, S; light green sphere, Cl; grey sphere, C; light grey sphere, H.

The [Ag_37_(S-Adm)_25_Cl_1_]^+^ nanocluster was obtained when the DPPB ligand was introduced to the reaction (Fig. 2B and S18†). The [Ag_37_(S-Adm)_25_Cl_1_]^+^ cluster entities are crystallized in a monoclinic crystal system with a *P*2_1_/*c* space group. The [Ag_37_(S-Adm)_25_Cl_1_]^+^ nanocluster comprises a planar Ag_18_Cl_1_ core that is capped by a ring-like Ag_6_(S-Adm)_6_ motif and a S-Adm on the same face (Fig. 7A–D). Then, three dimeric Ag_2_(S-Adm)_3_ motif structures wrapped the side of the planar Ag_18_Cl_1_ core, giving rise to an Ag_30_(S-Adm)_16_Cl_1_ structure (Fig. 7E and F). Finally, another three dimeric Ag_2_(S-Adm)_3_ motifs that are anchored by an Ag atom stabilize another face of the planar Ag_18_Cl_1_ core (*i.e.*, the opposite face with the Ag_6_(S-Adm)_6_ motif), forming the Ag_37_(SR)_25_Cl_1_ framework (Fig. 7G–I). Similar to [Ag_34_(S-Adm)_18_(DPPP)_3_Cl_4_]^2+^, the overall structure of [Ag_37_(S-Adm)_25_Cl_1_]^+^ nanocluster is triple axisymmetric with a *C*_3_ symmetry axis passing through the Cl atom on the Ag_18_Cl_1_ core and the anchoring Ag atom on the surface (Fig. 7G and K).

Similar to the structure of [Ag_34_(S-Adm)_18_(DPPP)_3_Cl_4_]^2+^, the kernel of [Ag_37_(S-Adm)_25_Cl_1_]^+^ also contains a surface Cl capping the core structure, making up an Ag_3_–Cl tetrahedral subunit. In the Ag_13_Cl_1_ core of [Ag_34_(S-Adm)_18_(DPPP)_3_Cl_4_]^2+^, no Ag–Ag interaction was observed within the Ag_3_–Cl subunit, and the average distances of Ag–Ag and Ag–Cl are 3.375 and 2.693 Å, respectively (Fig. 6). By comparison, the Ag_3_–Cl tetrahedral subunit in the Ag_18_Cl_1_ core of [Ag_37_(S-Adm)_25_Cl_1_]^+^ is more compact with average Ag–Ag and Ag–Cl distances of 2.904 and 2.540 Å, respectively (Fig. 7).

The Ag–Cl and Ag–S interactions in these obtained Ag nanoclusters were then compared. As shown in Table S9,† the bond lengths of surface Ag–Cl (or surface Ag–S) are much longer than those of kernel Ag–Cl (or kernel Ag–S). Besides, the bond lengths of Ag–S are much shorter than those of Ag–Cl, which resulted from the different interactions between Ag–S and Ag–Cl.

### Characterization studies and optical absorptions

The ESI-MS measurement was performed to confirm the compositions and determine the valence states of the obtained silver nanoclusters. As shown in Fig. S19–S25,† the mass peaks at 5217.72, 4180.39, 5708.24, 4028.85, and 8208.69 Da confirmed the compositions of the crystal structures of these silver nanoclusters, and demonstrated their valence states to be [Ag_52_(S-Adm)_28_Cl_4_]^2+^, [Ag_36_(S-Adm)_26_S_4_]^2+^, [Ag_25_(S-Adm)_18_]^−^, [Ag_34_(S-Adm)_18_(DPPP)_3_Cl_4_]^2+^, and [Ag_37_(S-Adm)_25_Cl_1_]^+^, respectively. The compositions of [Ag_1_(DPPE)_2_]^+^ and [Ag_3_(S-Adm)_2_(DPPH)_2_]^+^ counterions of [Ag_25_(S-Adm)_18_]^−^ were also verified (Fig. S22 and S25†). Besides, the presence of “SbF_6_^−^” counterions of [Ag_36_(S-Adm)_26_S_4_]^2+^ and [Ag_34_(S-Adm)_18_(DPPP)_3_Cl_4_]^2+^ nanoclusters was also confirmed by ESI-MS (Fig. S21 and S23†).

According to the valence states of these nanoclusters, their nominal electron counts were determined:^59^ 52(Ag) − 28(SR) − 4(Cl) − 2(charge) = 18e for [Ag_52_(S-Adm)_28_Cl_4_]^2+^, 36(Ag) − 26(SR) − 4 × 2(S) − 2(charge) = 0e for [Ag_36_(S-Adm)_26_S_4_]^2+^, 25(Ag) − 18(SR) + 1(charge) = 8e for [Ag_25_(S-Adm)_18_]^−^, 34(Ag) − 18(SR) − 4(Cl) − 2(charge) = 10e for [Ag_34_(S-Adm)_18_(DPPP)_3_Cl_4_]^2+^, and 37(Ag) − 25(SR) − 1(Cl) − 1(charge) = 10e for [Ag_37_(S-Adm)_25_Cl_1_]^+^. Compared with other silver nanoclusters, the [Ag_36_(S-Adm)_26_S_4_]^2+^ nanocluster had no nominal electron counts, and could be recognized as a nanocluster complex. Such a conclusion, *i.e.*, [Ag_36_(S-Adm)_26_S_4_]^2+^ was a nanocluster complex, corresponded to that the Ag_8_S_4_ kernel in this nanocluster was not a pure-metal kernel.

The optical absorptions of the obtained silver nanoclusters (dissolved in CH_2_Cl_2_) were then measured (Fig. S26†). [Ag_52_(S-Adm)_28_Cl_4_]^2+^ displayed two intense absorptions at 500 and 620 nm, and two shoulder bands at 375 and 670 nm (Fig. S26A†). [Ag_36_(S-Adm)_26_S_4_]^2+^ exhibited several shoulder bands at 500, 625, and 690 nm (Fig. S26B†). [Ag_34_(S-Adm)_18_(DPPP)_3_Cl_4_]^2+^ displayed two obvious absorptions at 420 and 575 nm, and two shoulder bands at 380 and 460 nm (Fig. S26D†). [Ag_37_(S-Adm)_25_Cl_1_]^+^ showed a series of absorptions at 345, 435, 565, 730, and 895 nm (Fig. S26E†). [Ag_25_(S-Adm)_18_]^−^ displayed two intense bands at 500 and 690 nm, showing ∼10 nm red-shift relative to the corresponding bands of [Ag_25_(S-PhMe_2_)_18_]^−^ (Fig. S26C and F†).^58^ Such a red-shift resulted from the ligand effect on nanocluster electronic structures, since the alternation of ligands of nanoclusters would affect their molecular orbital energy levels, embodied by their optical absorptions.^33,60–62^

## Conclusions

In summary, a “dual-level kinetic control” was exploited for synthesizing atomically precise silver nanoclusters and contributing to their high-yield preparation. Specifically, the introduction of phosphine ligands to the reaction retarded the reduction rate and accomplished the first-level kinetic control. Then, the cooling of the reaction further retarded the reduction rate and fulfilled the second-level kinetic control. A family of monodispersed Ag nanoclusters (including [Ag_25_(SR)_18_]^−^, [Ag_34_(SR)_18_(DPPP)_3_Cl_4_]^2+^, [Ag_36_(SR)_26_S_4_]^2+^, [Ag_37_(SR)_25_Cl_1_]^+^, and [Ag_52_(SR)_28_Cl_4_]^2+^) were controllably synthesized and structurally determined. The developed “dual-level kinetic control” in this work might potentially act as a powerful tool for the preparation of more nanoclusters with unprecedented atomic structures.

## Data availability

All the data supporting this article have been included in the main text and the ESI.†

## Author contributions

X. W. and C. X. carried out experiments and analyzed the data. H. L. assisted with the analysis. X. K. and M. Z. designed the project, analyzed the data, and wrote the manuscript.

## Conflicts of interest

There are no conflicts to declare.

## Supplementary Material

SC-013-D2SC01016J-s001

SC-013-D2SC01016J-s002
