# Supplementary material for: Fabrication of a family of atomically precise silver nanoclusters via dual-level kinetic control
Source: Chem Sci. 2022 Apr 10;13(19):5531–8. doi: 10.1039/d2sc01016j (PMC9116368; doi:10.1039/d2sc01016j)
Supplement: SC-013-D2SC01016J-s001 [file SC-013-D2SC01016J-s001.pdf]

Electronic Supplementary Material (ESI) for Chemical Science.
This journal is © The Royal Society of Chemistry 2022

Supporting Information

Fabrication of a Family of Atomically Precise Silver Nanoclusters via Dual-Level Kinetic Control

Xiao Wei,‡ Chao Xu,‡ Hao Li, Xi Kang,* and Manzhou Zhu*

Department of Chemistry and Centre for Atomic Engineering of Advanced Materials, Key Laboratory of Structure and Functional Regulation of Hybrid Materials of Ministry of Education, Institutes of Physical Science and Information Technology and Anhui Province Key Laboratory of Chemistry for Inorganic/Organic Hybrid Functionalized Materials, Anhui University, Hefei, Anhui 230601, China.

‡X.W. and C.X. contributed equally.

*E-mails of corresponding authors: kangxi_chem@ahu.edu.cn (X.K.); zmz@ahu.edu.cn (M.Z.).

Notes: The authors declare no competing financial interest.

This PDF file includes:
Figs. S1-S26
Tables S1-S9

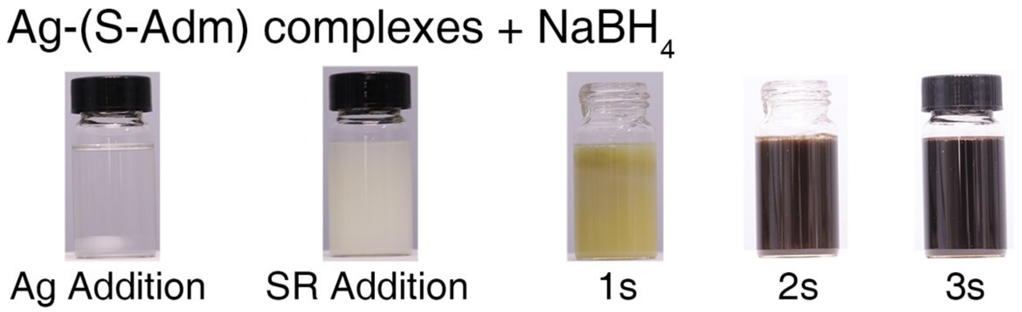

Fig. S1 Photos of the reduction of Ag-(S-Adm) complexes by $NaBH_4$ in CH_2Cl_2. After the introduction of $NaBH_4$, the solution turned black within three seconds, demonstrating the rapid reduction rate.

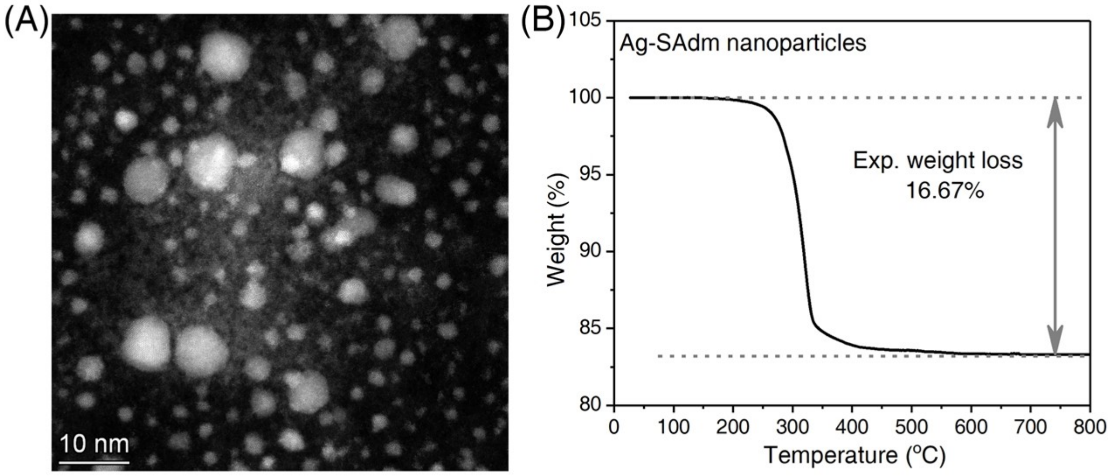

Fig. S2 (A) The HAADF-STEM image of the CH_2Cl_2 supernatant for reducing the Ag-(S-Adm) complexes by $NaBH_4$ (the reaction lasted for 12 hours after the reductant introduction). Polydisperse metal nanoparticles (from ~1 to ~10 nm) were observed. (B) TGA result of these polydisperse silver nanoparticles. The Ag-to-SAdm ratio of these nanoparticles was determined as 16.67%.

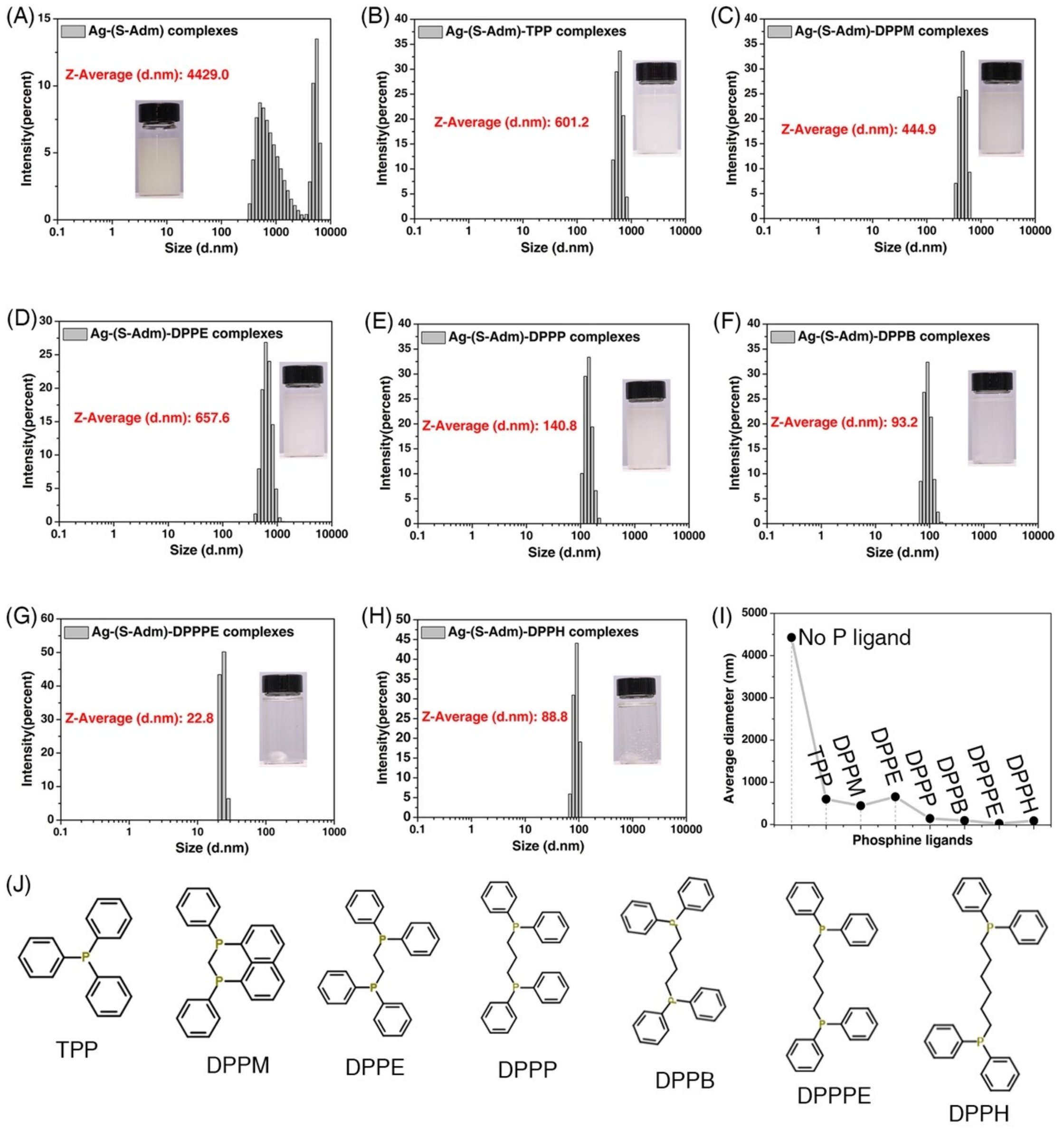

Fig. S3 DLS results of Ag-(S-Adm) or Ag-(S-Adm)-PR complexes. (A) The average diameter of the Ag-(S-Adm) complexes in CH_2Cl_2 was determined as 4429.0 nm. (B) The average diameter of the Ag-(S-Adm)-TPP complexes in CH_2Cl_2 was determined as 601.2 nm. (C) The average diameter of the Ag-(S-Adm)-DPPM complexes in CH_2Cl_2 was determined as 444.9 nm. (D) The average diameter of the Ag-(S-Adm)-DPPE complexes in CH_2Cl_2 was determined as 657.6 nm. (E) The average diameter of the Ag-(S-Adm)-DPPP complexes in CH_2Cl_2 was determined as 140.8 nm. (F) The average diameter of the Ag-(S-Adm)-DPPB complexes in CH_2Cl_2 was determined as 93.92 nm. (G) The average diameter of the Ag-(S-Adm)-DPPPE complexes in CH_2Cl_2 was determined as 22.8 nm. (H) The average diameter of the Ag-(S-Adm)-DPPH complexes in CH_2Cl_2 was determined as 88.8 nm. (I) Comparison of average diameters of Ag-(S-Adm) or Ag-(S-Adm)-PR complexes. (J) Structures of different phosphine ligands, including TPP, DPPM, DPPE, DPPP, DPPB, DPPPE, and DPPH.

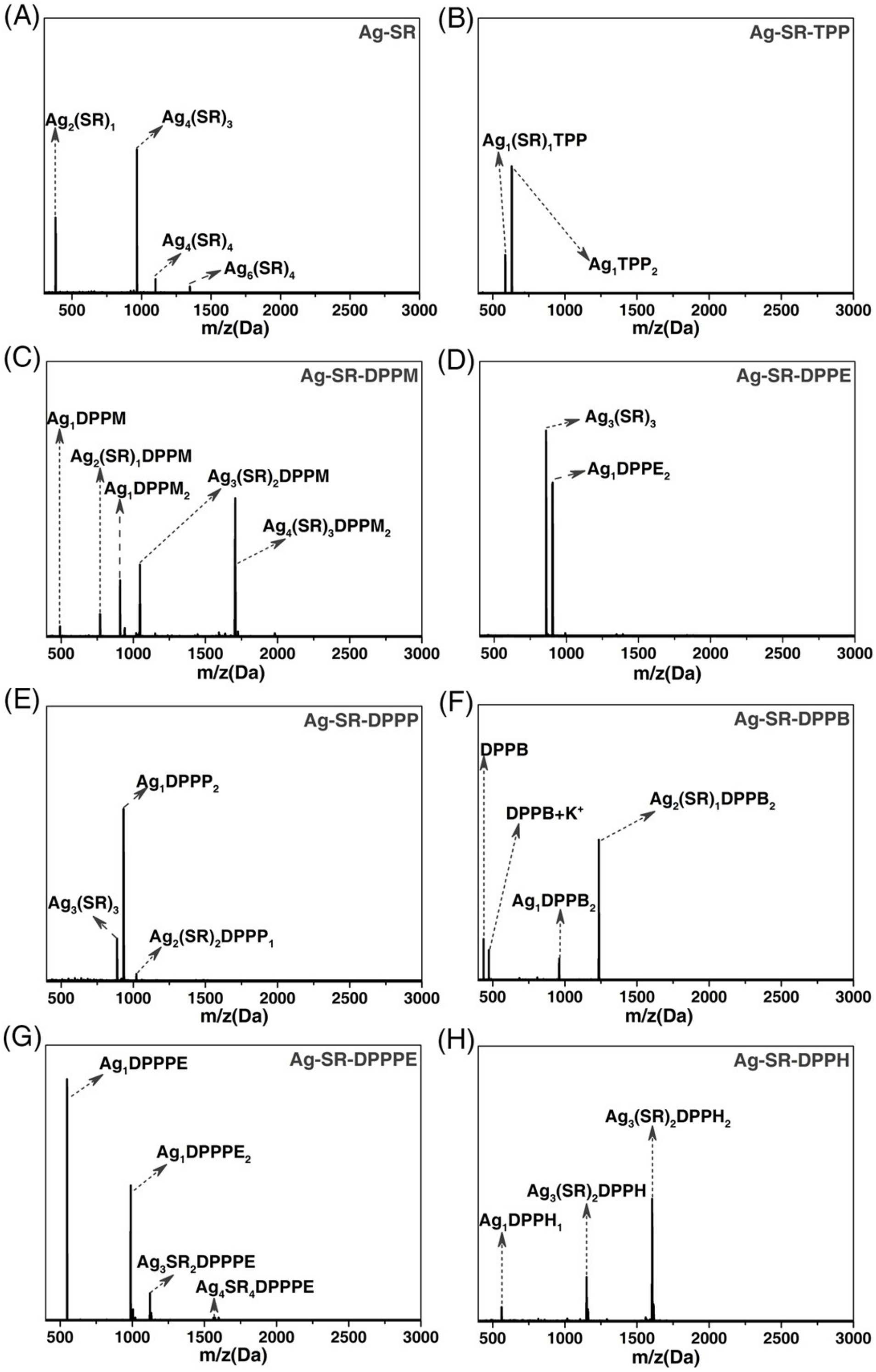

Fig. S4 ESI-MS results of Ag-(S-Adm) or Ag-(S-Adm)-PR complexes in the positive mode. (A) ESI-MS results of Ag-(S-Adm) complexes. (B) ESI-MS results of Ag-(S-Adm)-TPP complexes. (C) ESI-MS results of Ag-(S-Adm)-DPPM complexes. (D) ESI-MS results of Ag-(S-Adm)-DPPE complexes. (E) ESI-MS results of Ag-(S-Adm)-DPPP complexes. (F) ESI-MS results of Ag-(S-Adm)-DPPB complexes. (G) ESI-MS results of Ag-(S-Adm)-DPPPE complexes. (H) ESI-MS results of Ag-(S-Adm)-DPPH complexes.

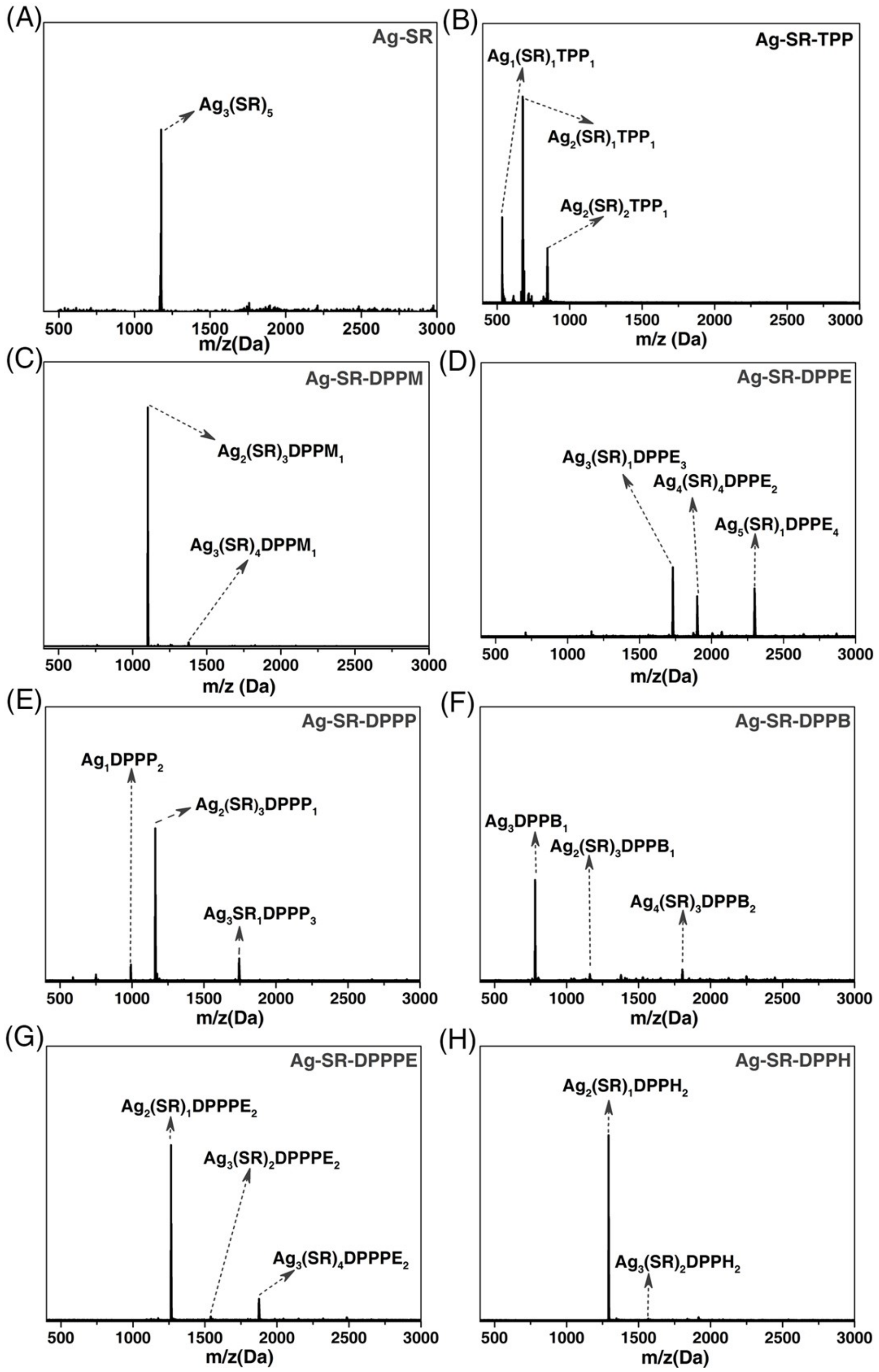

Fig. S5 ESI-MS results of Ag-(S-Adm) or Ag-(S-Adm)-PR complexes in the negative mode. (A) ESI-MS results of Ag-(S-Adm) complexes. (B) ESI-MS results of Ag-(S-Adm)-TPP complexes. (C) ESI-MS results of Ag-(S-Adm)-DPPM complexes. (D) ESI-MS results of Ag-(S-Adm)-DPPE complexes. (E) ESI-MS results of Ag-(S-Adm)-DPPP complexes. (F) ESI-MS results of Ag-(S-Adm)-DPPB complexes. (G) ESI-MS results of Ag-(S-Adm)-DPPPE complexes. (H) ESI-MS results of Ag-(S-Adm)-DPPH complexes.

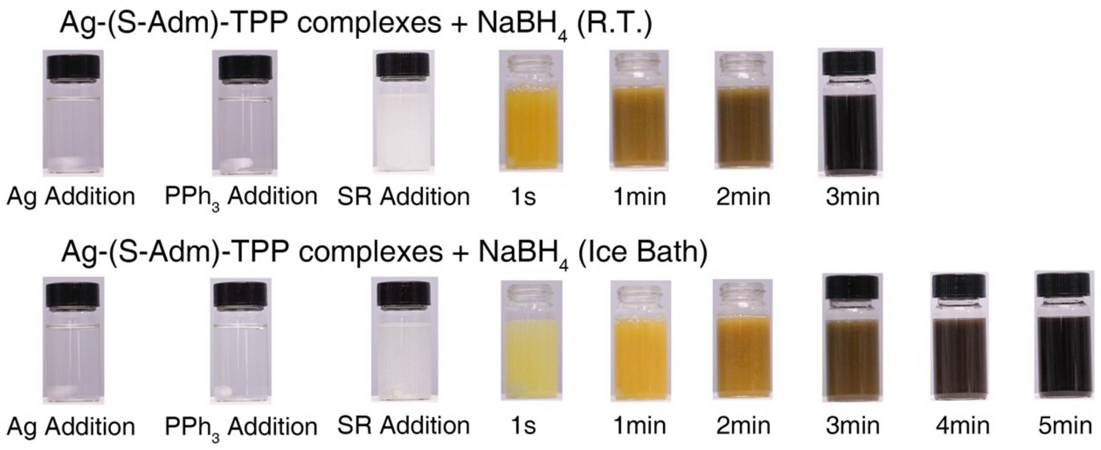

Fig. S6 Photos of the reduction of Ag-(S-Adm)-TPP complexes by $NaBH_4$ in CH_2Cl_2 at different times at room temperature or under ice bath.

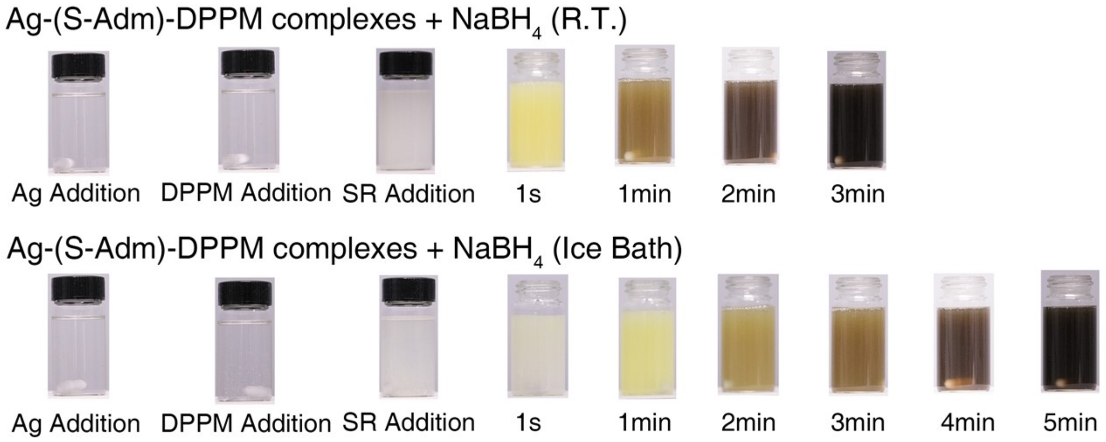

Fig. S7 Photos of the reduction of Ag-(S-Adm)-DPPM complexes by $NaBH_4$ in CH_2Cl_2 at different times at room temperature or under ice bath.

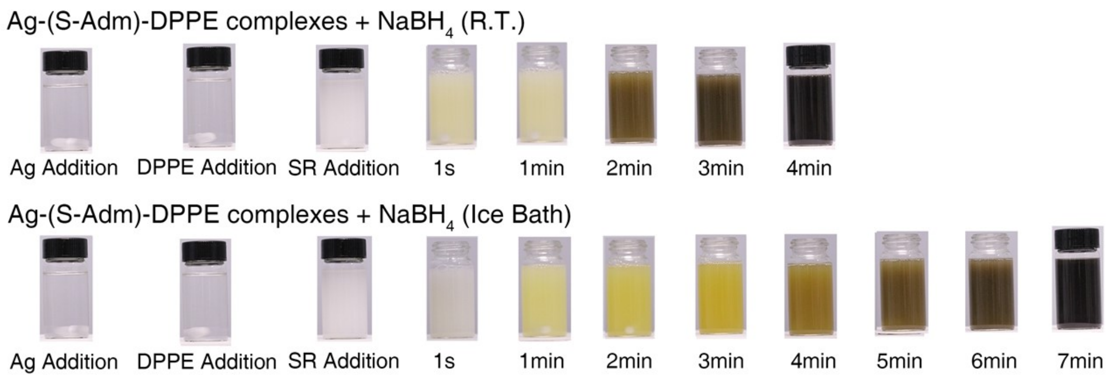

Fig. S8 Photos of the reduction of Ag-(S-Adm)-DPPE complexes by $NaBH_4$ in CH_2Cl_2 at different times at room temperature or under ice bath.

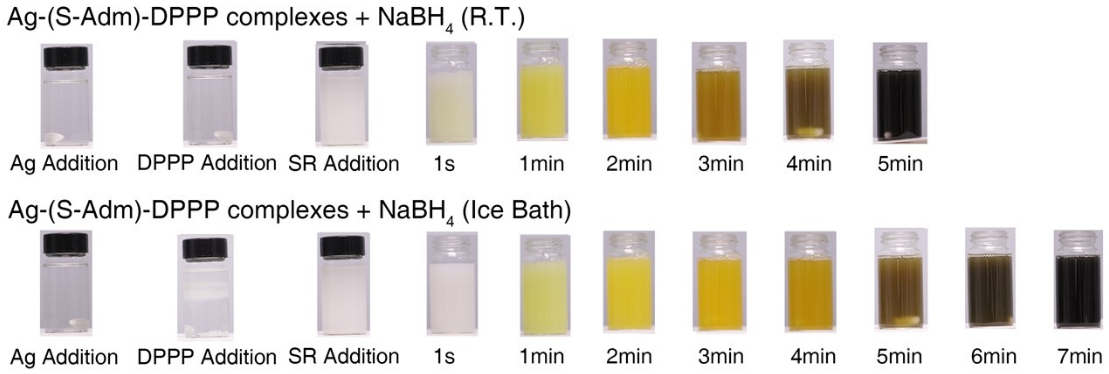

Fig. S9 Photos of the reduction of Ag-(S-Adm)-DPPP complexes by $NaBH_4$ in CH_2Cl_2 at different times at room temperature or under ice bath.

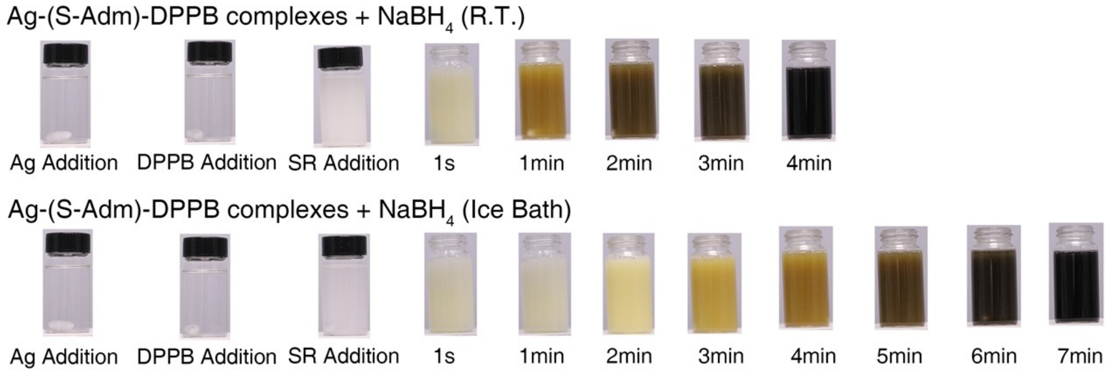

Fig. S10 Photos of the reduction of Ag-(S-Adm)-DPPB complexes by $NaBH_4$ in CH_2Cl_2 at different times at room temperature or under ice bath.

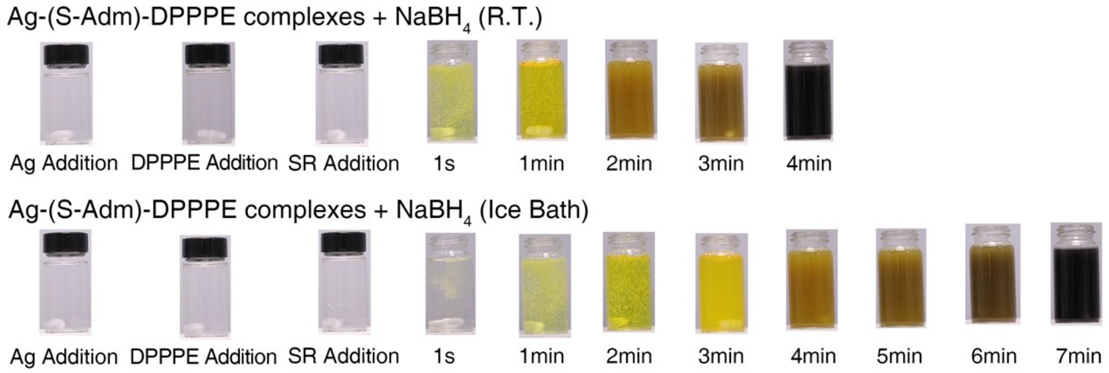

Fig. S11 Photos of the reduction of Ag-(S-Adm)-DPPPE complexes by $NaBH_4$ in CH_2Cl_2 at different times at room temperature or under ice bath.

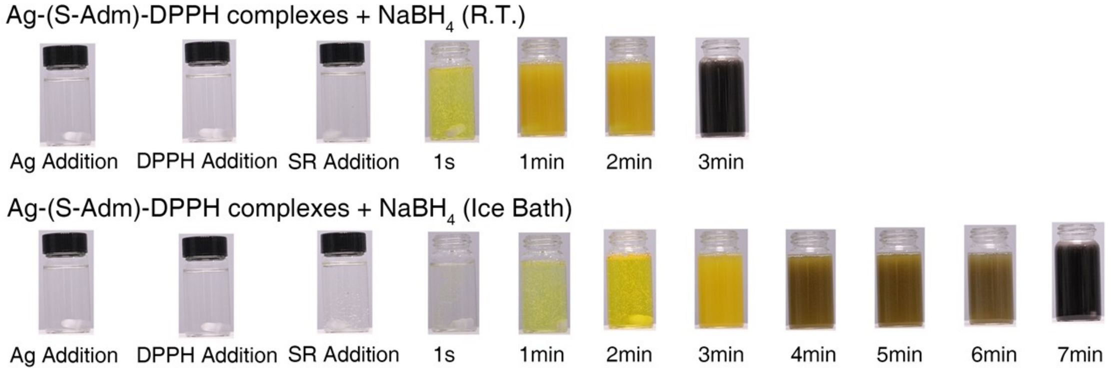

Fig. S12 Photos of the reduction of Ag-(S-Adm)-DPPH complexes by $NaBH_4$ in CH_2Cl_2 at different times at room temperature or under ice bath.

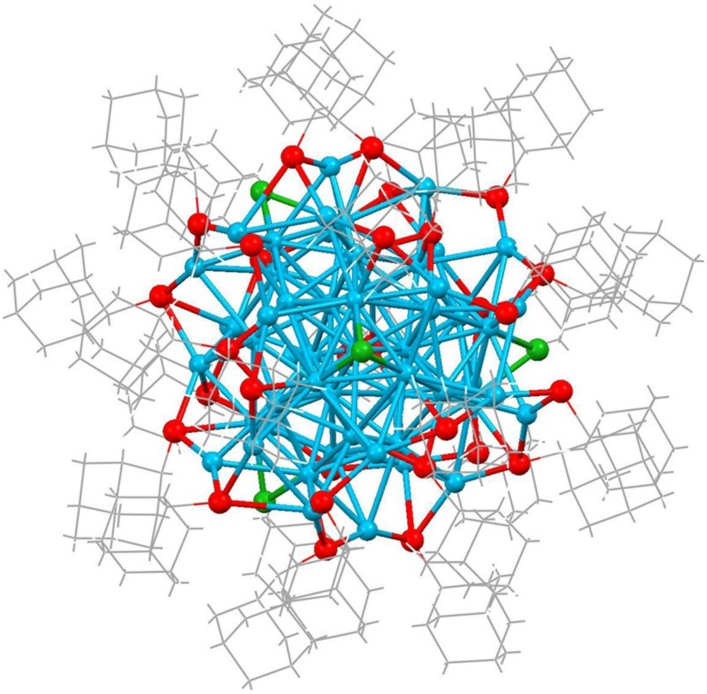

Fig. S13 Overall structure of the $[Ag_{52}(S\text{-}Adm)_{28}Cl_4]^{2+}$ nanocluster. Color legends: light blue sphere, Ag; red sphere, S; green sphere, Cl; grey sphere, C; white sphere, H.

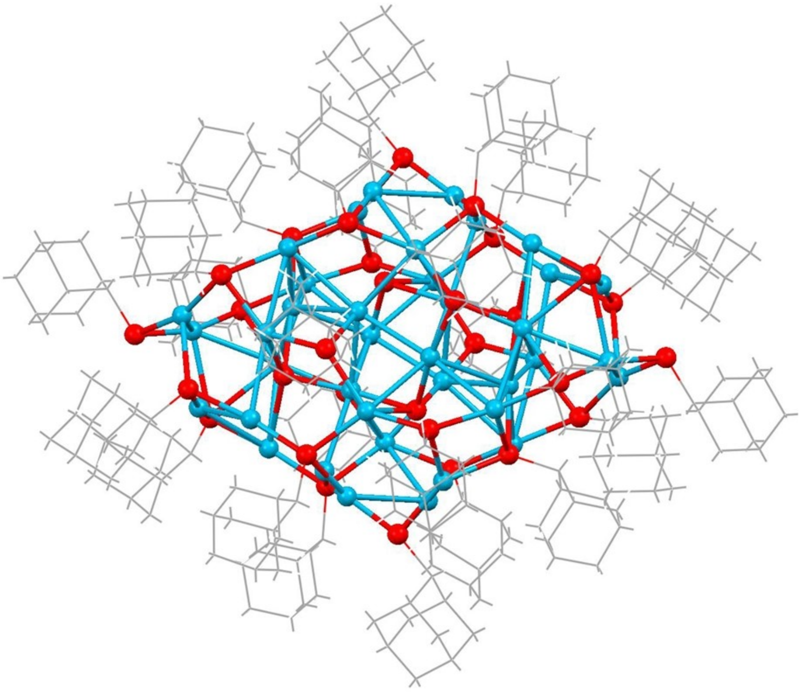

Fig. S14 Overall structure of the $[Ag_{36}(S\text{-}Adm)_{26}S_4]^{2+}$ nanocluster. Color legends: light blue sphere, Ag; red sphere, S; grey sphere, C; white sphere, H.

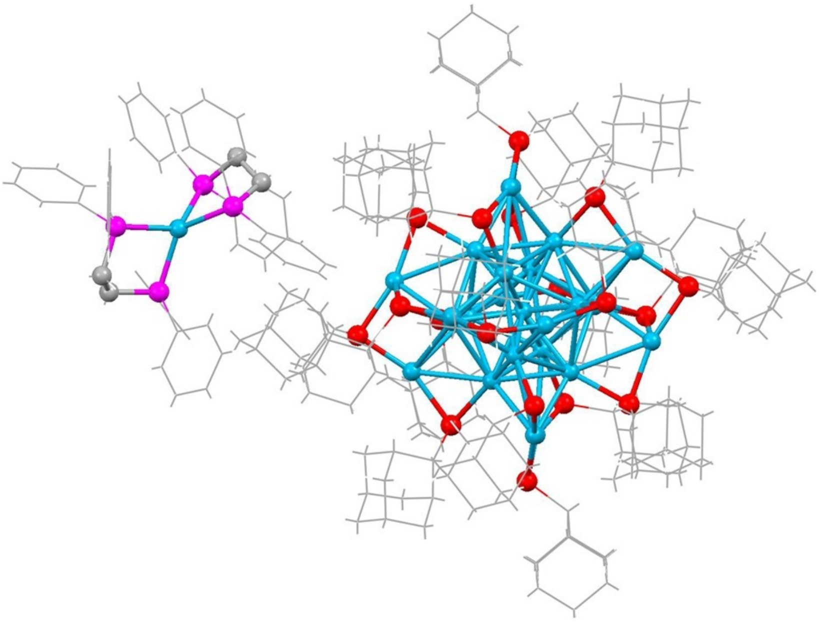

Fig. S15 Overall structure of the $[Ag_{25}(S\text{-}Adm)_{18}]^{-}[Ag_1(DPPE)_2]^{+}$ nanocluster. Color legends: light blue sphere, Ag; red sphere, S; magenta sphere, P; grey sphere, C; white sphere, H.

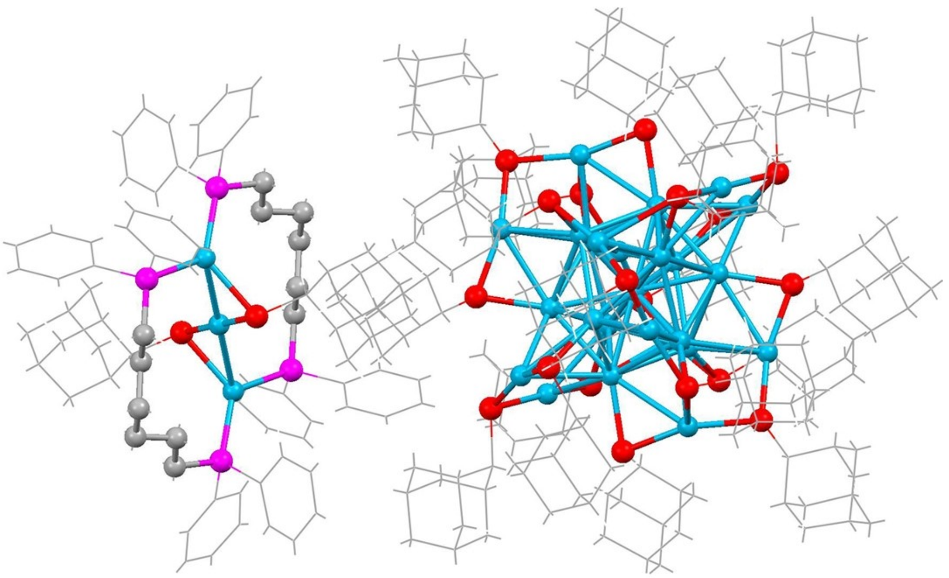

Fig. S16 Overall structure of the $[Ag_{25}(S\text{-}Adm)_{18}]^{-}[Ag_3(DPPH)_2(S\text{-}Adm)_2]^{+}$ nanocluster. Color legends: light blue sphere, Ag; red sphere, S; magenta sphere, P; grey sphere, C; white sphere, H.

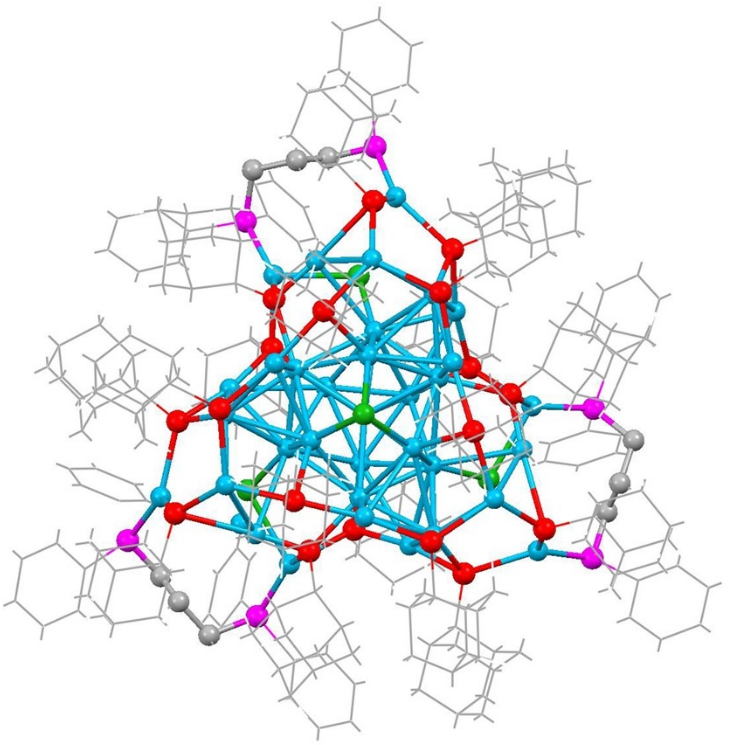

Fig. S17 Overall structure of the $[Ag_{34}(S\text{-}Adm)_{18}(DPPP)_3Cl_4]^{2+}$ nanocluster. Color legends: light blue sphere, Ag; red sphere, S; magenta sphere, P; green sphere, Cl; grey sphere, C; white sphere, H.

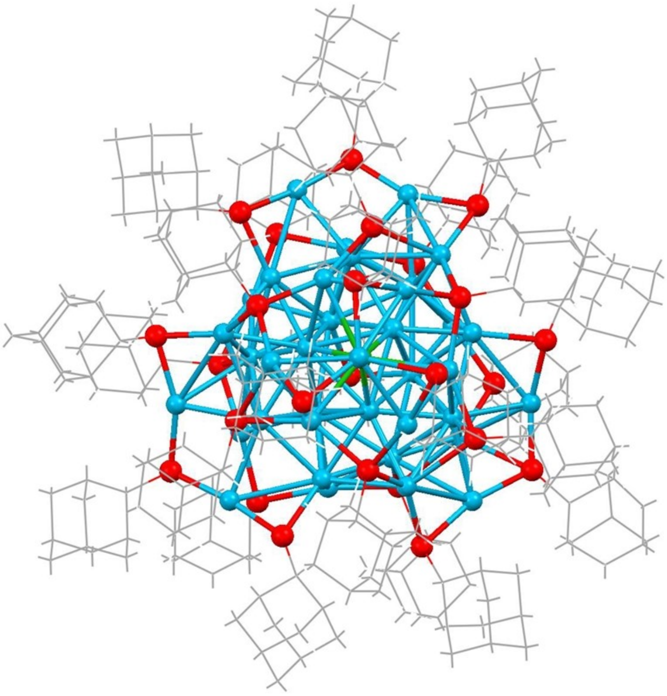

Fig. S18 Overall structure of the $[Ag_{37}(S\text{-}Adm)_{25}Cl_1]^+$ nanocluster. Color legends: light blue sphere, Ag; red sphere, S; green sphere, Cl; grey sphere, C; white sphere, H.

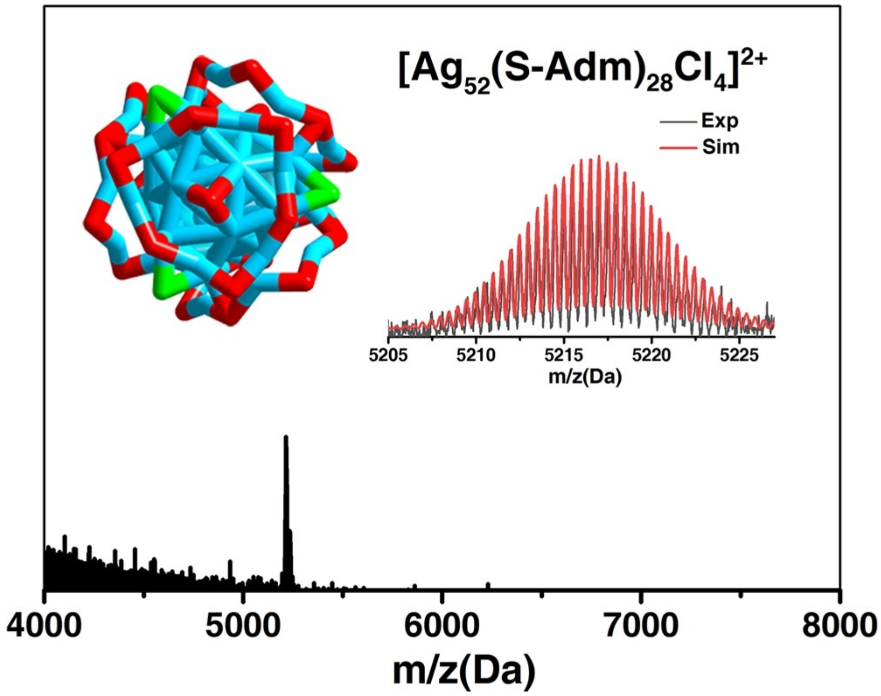

Fig. S19 ESI-MS result of the $[Ag_{52}(S\text{-}Adm)_{28}Cl_4]^{2+}$ nanocluster. Insets: the framework of the nanocluster and the comparison of the experiment (black line) and the simulated (red line) isotopic distributions. This $[Ag_{52}(S\text{-}Adm)_{28}Cl_4]^{2+}$ nanocluster was obtained by reducing the Ag-SR-TPP complexes by $NaBH_4$.

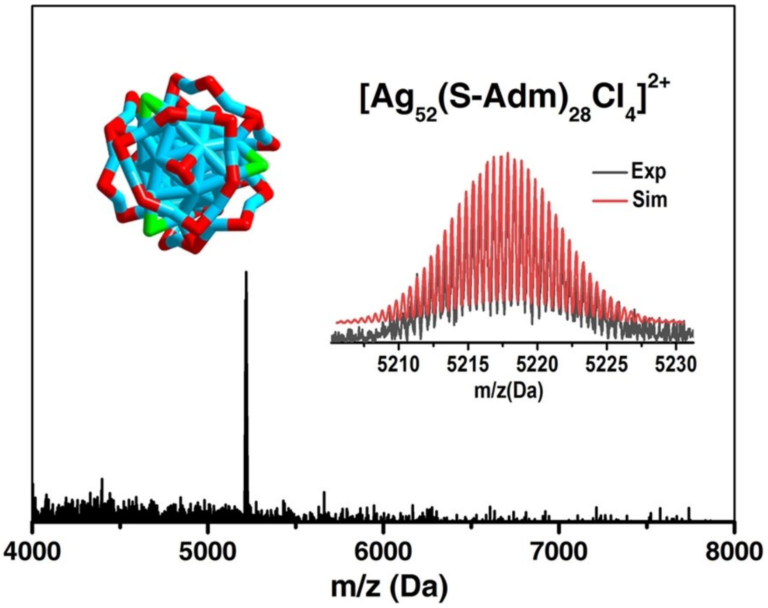

Fig. S20 ESI-MS result of the $[Ag_{52}(S\text{-}Adm)_{28}Cl_4]^{2+}$ nanocluster. Insets: the framework of the nanocluster and the comparison of the experiment (black line) and the simulated (red line) isotopic distributions. This $[Ag_{52}(S\text{-}Adm)_{28}Cl_4]^{2+}$ nanocluster was obtained by reducing the Ag-SR-DPPPE complexes by $NaBH_4$.

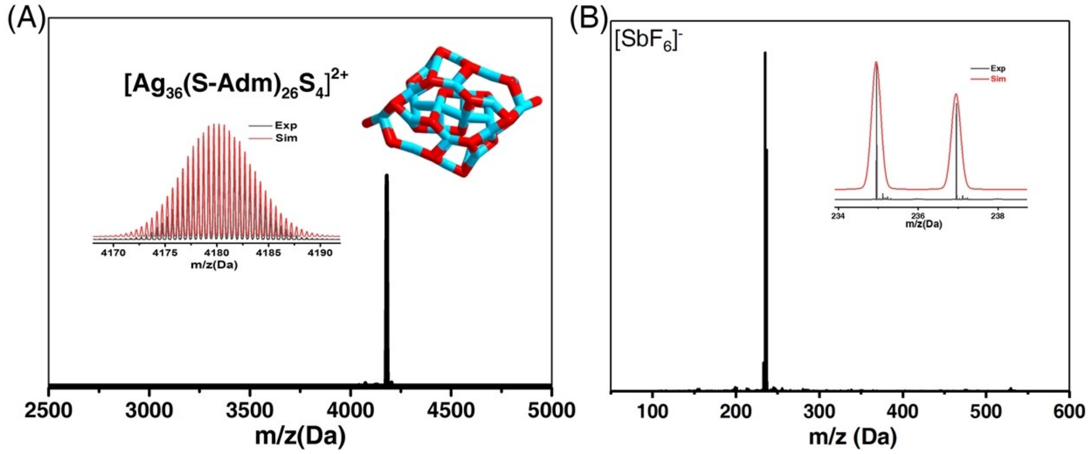

Fig. S21 (A) ESI-MS result of the $[Ag_{36}(S\text{-}Adm)_{26}S_4]^{2+}$ nanocluster. Insets: the framework of the nanocluster and the comparison of the experiment (black line) and the simulated (red line) isotopic distributions. This $[Ag_{36}(S\text{-}Adm)_{26}S_4]^{2+}$ nanocluster was obtained by reducing the Ag-SR-DPPM complexes by $NaBH_4$. (B) ESI-MS result of the SbF_6^- counterion.

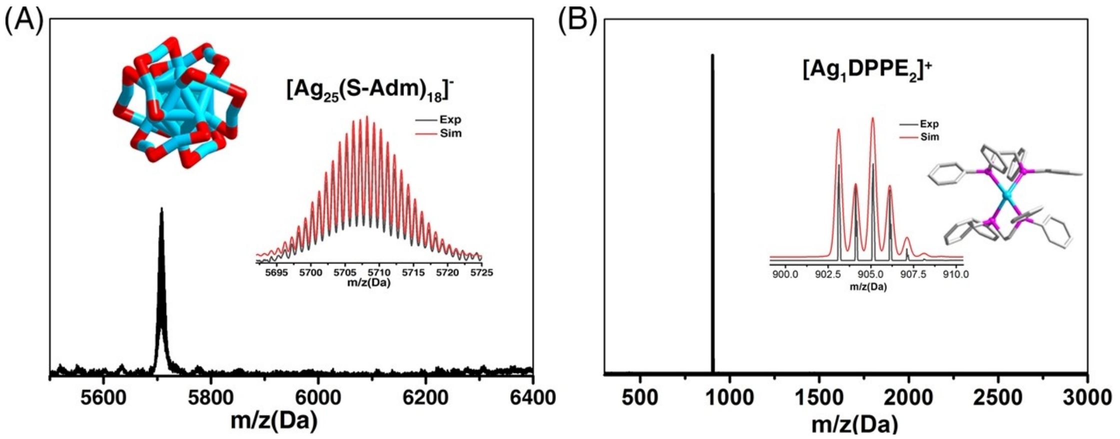

Fig. S22 (A) ESI-MS result of the $[Ag_{25}(S\text{-}Adm)_{18}]^-$ nanocluster. (B) ESI-MS result the $[Ag_1(DPPE)_2]^+$ complex. Insets: the framework of the nanocluster and the comparison of the experiment (black line) and the simulated (red line) isotopic distributions. This $[Ag_{25}(S\text{-}Adm)_{18}]^-[Ag_1(DPPE)_2]^+$ nanocluster was obtained by reducing the Ag-SR-DPPE complexes by $NaBH_4$.

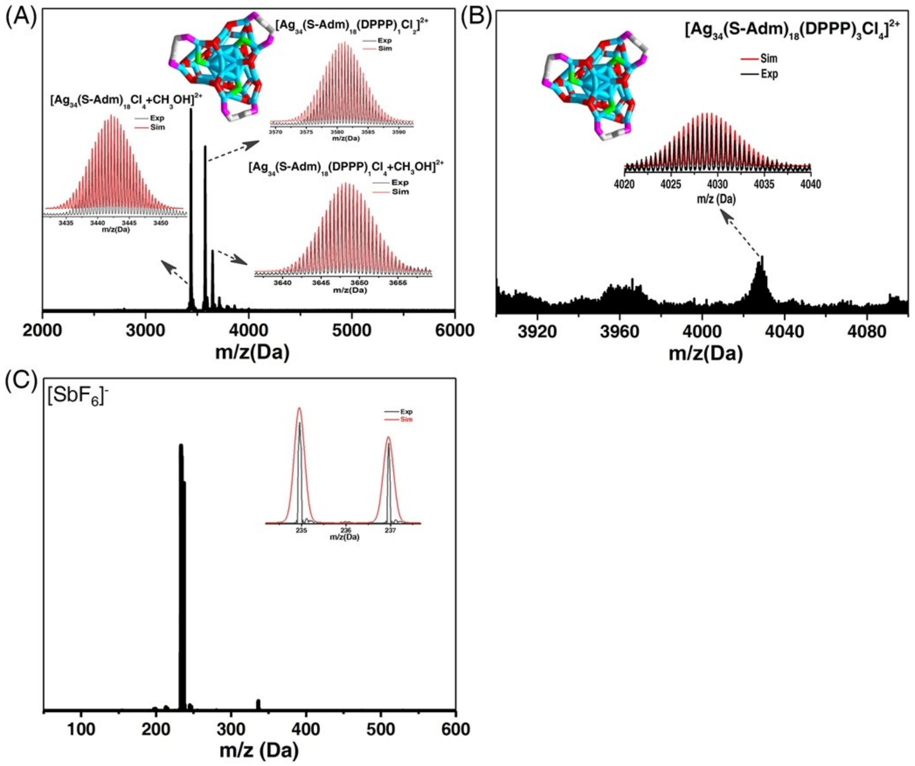

Fig. S23 (A, B) ESI-MS result of the $[Ag_{34}(S\text{-}Adm)_{18}(DPPP)_3Cl_4]^{2+}$ nanocluster. Probably because the DPPP and Cl ligands were easily dissociated from the $Ag_{34}(S\text{-}Adm)_{18}(DPPP)_3Cl_4$ nanocluster, several incomplete mass signals were detected, including $[Ag_{34}(S\text{-}Adm)_{18}Cl_4 + CH_3OH]^{2+}$, $[Ag_{34}(S\text{-}Adm)_{18}(DPPP)_1Cl_2]^{2+}$, and $[Ag_{34}(S\text{-}Adm)_{18}(DPPP)_1Cl_4 + CH_3OH]^{2+}$ (Figure S23A), whereas the molecular ion peak (i.e., $[Ag_{34}(S\text{-}Adm)_{18}(DPPP)_3Cl_4]^{2+}$) was weak (Figure S23B). Insets: the framework of the nanocluster and the comparison of the experiment (black line) and the simulated (red line) isotopic distributions. This $[Ag_{34}(S\text{-}Adm)_{18}(DPPP)_3Cl_4]^{2+}$ nanocluster was obtained by reducing the Ag-SR-DPPP complexes by $NaBH_4$. (C) ESI-MS result of the SbF_6^- counterion.

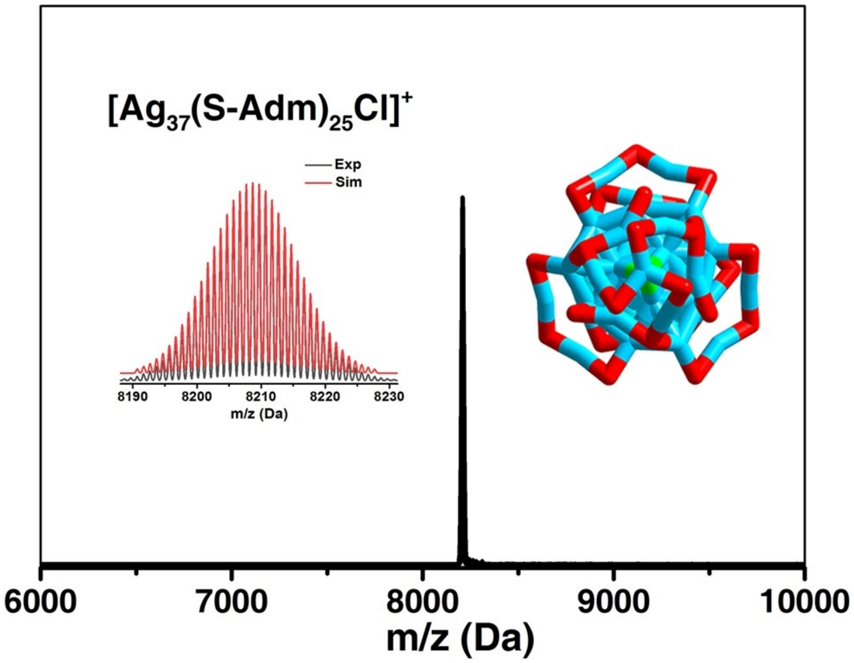

Fig. S24 ESI-MS result of the $[Ag_{37}(S\text{-}Adm)_{25}Cl_1]^+$ nanocluster. Insets: the framework of the nanocluster and the comparison of the experiment (black line) and the simulated (red line) isotopic distributions. This $[Ag_{37}(S\text{-}Adm)_{25}Cl_1]^+$ nanocluster was obtained by reducing the Ag-SR-DPPB complexes by $NaBH_4$.

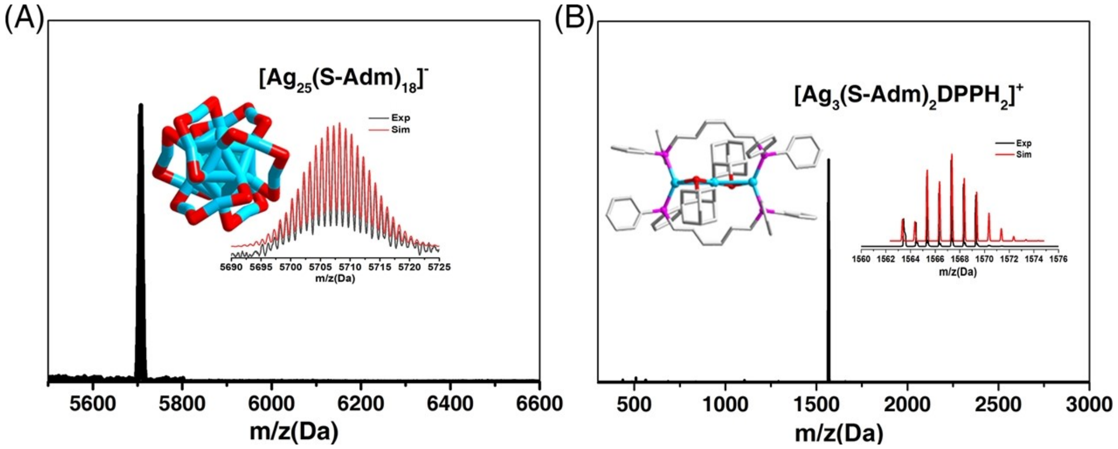

Fig. S25 (A) ESI-MS result of the $[Ag_{25}(S\text{-}Adm)_{18}]^-$ nanocluster. (B) ESI-MS result the $[Ag_3(S\text{-}Adm)_2(DPPH)_2]^+$ complex. Insets: the framework of the nanocluster and the comparison of the experiment (black line) and the simulated (red line) isotopic distributions. This $[Ag_{25}(S\text{-}Adm)_{18}]^-$ $[Ag_3(S\text{-}Adm)_2(DPPH)_2]^+$ nanocluster was obtained by reducing the Ag-SR-DPPH complexes by $NaBH_4$.

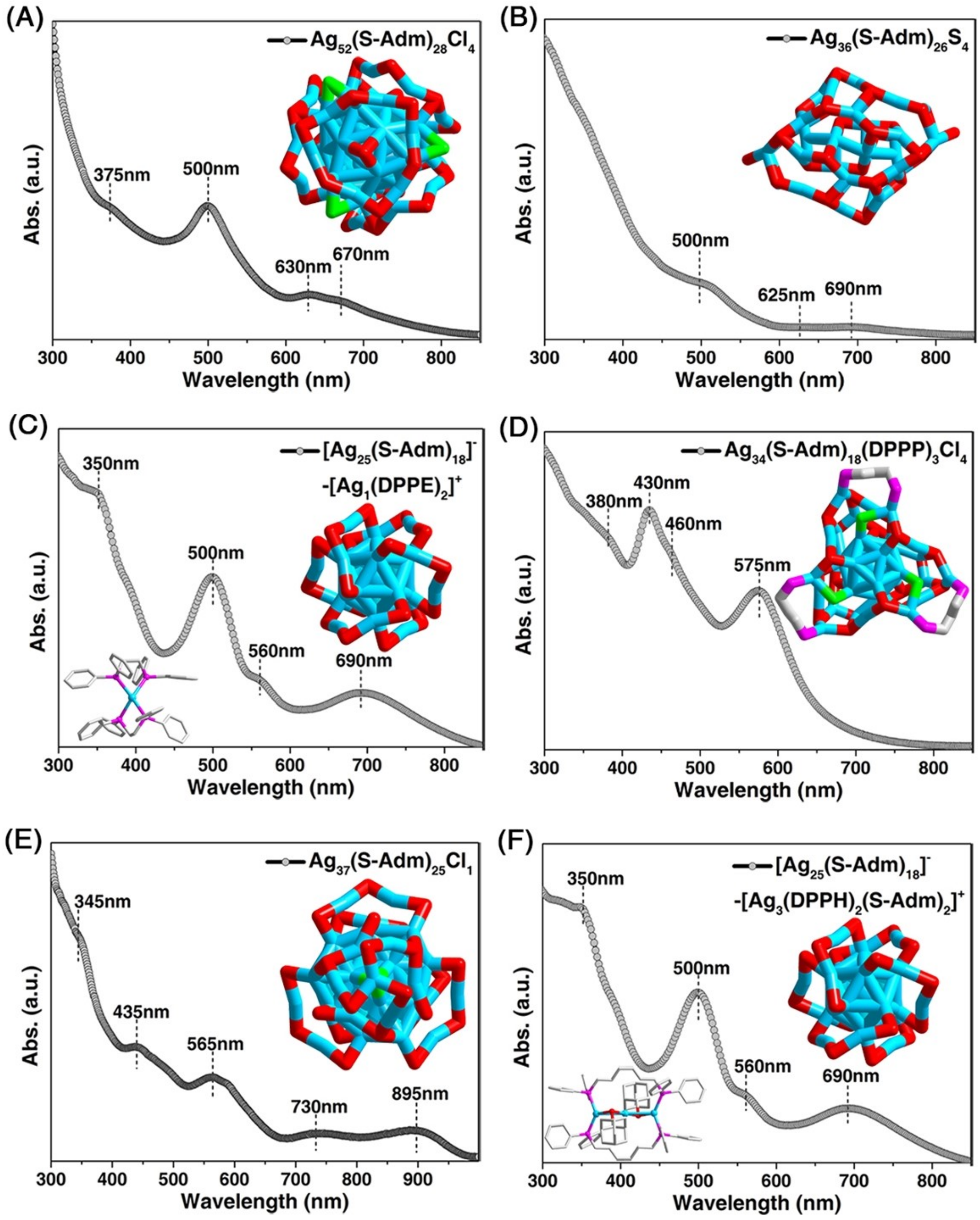

Fig. S26 Optical absorptions of the obtained silver nanoclusters (dissolved in CH_2Cl_2). (A) UV-vis spectrum of $[Ag_{52}(S\text{-}Adm)_{28}Cl_4]^{2+}$. (B) UV-vis spectrum of $[Ag_{36}(S\text{-}Adm)_{26}S_4]^{2+}$. (C) UV-vis spectrum of $[Ag_{25}(S\text{-}Adm)_{18}]^-[Ag_1(DPPE)_2]^+$. (D) UV-vis spectrum of $[Ag_{34}(S\text{-}Adm)_{18}(DPPP)_3Cl_4]^{2+}$. (E) UV-vis spectrum of $[Ag_{37}(S\text{-}Adm)_{25}Cl_1]^+$. (F) UV-vis spectrum of $[Ag_{25}(S\text{-}Adm)_{18}]^-[Ag_3(S\text{-}Adm)_2(DPPH)_2]^+$.

Table S1. Crystal data and structure refinement for the $[Ag_{52}(S\text{-}Adm)_{28}Cl_4]^{2+}$ nanocluster (prepared in the presence of TPP). The CCDC number of the $[Ag_{52}(S\text{-}Adm)_{28}Cl_4]^{2+}$ nanocluster (prepared in the presence of TPP) is 2094270.

Crystal system	orthorhombic
Space group	P c c n
a/Å	22.7708(5)
b/Å	37.8932(8)
c/Å	47.1176(9)
α/°	90
β/°	90
γ/°	90
Volume/Å^3	40655.8(15)
Z	4
ρcalcg/cm^3	1.705
μ/mm^{-1}	21.409
F(000)	20240
Radiation	CuKα (λ = 1.54186)
Index ranges	$-26 \le h \le 20$, $-43 \le k \le 20$, $-54 \le l \le 54$
θ range (°)	2.940 – 62.499
Measured reflections and unique reflections	151947 / 31624 (R_{int} =0.0427)
Goodness-of-fiton F^2	1.047
Largest diff. peak/hole / e Å^{-3}	2.9/-2.5
Final R indexes [I>=2σ (I)]	R_1 = 0.0514, wR_2 = 0.1423
Final R indexes [all data]	R_1 = 0.0636, wR_2 = 0.1371

Table S2. Crystal data and structure refinement for the $[Ag_{36}(S\text{-}Adm)_{28}S_4](SbF_6)_2$ nanocluster. The CCDC number of $[Ag_{36}(S\text{-}Adm)_{28}S_4](SbF_6)_2$ is 2094271.

Crystal system	monoclinic
Space group	P 21/n
a/Å	22.2769(4)
b/Å	29.4661(4)
c/Å	24.0645(4)
α/°	90
β/°	95.0370(10)
γ/°	90
Volume/Å^3	15735.3(4)
Z	2
ρcalcg/cm^3	1.864
μ/mm^{-1}	21.085
F(000)	8664
Radiation	CuKα (λ = 1.54186)
Index ranges	-25 ≤ h ≤ 21, -29 ≤ k ≤ 33, -47 ≤ l ≤ 22
θ range (°)	3.521 – 62.499
Measured reflections and unique reflections	49442 / 24374 (R_{int} =0.0470)
Goodness-of-fiton F^2	1.029
Largest diff. peak/hole / e Å^{-3}	2.8/-4.9
Final R indexes [I>=2σ (I)]	R_1 = 0.0909, wR_2 = 0.2693
Final R indexes [all data]	R_1 = 0.1085, wR_2 = 0.2502

Table S3. Crystal data and structure refinement for the $[Ag_{25}(S\text{-}Adm)_{18}][Ag_1(DPPE)_2]$ nanocluster. The CCDC number of $[Ag_{25}(S\text{-}Adm)_{18}][Ag_1(DPPE)_2]$ is 2094272.

Crystal system	triclinic
Space group	P -1
a/Å	20.263(2)
b/Å	20.999(2)
c/Å	34.609(3)
α/°	90.312(8)
β/°	103.772(8)
γ/°	116.638(7)
Volume/$Å^3$	12677(2)
Z	2
ρcalcg/cm^3	1.732
μ/mm^{-1}	17.656
F(000)	6560
Radiation	CuKα (λ = 1.54186)
Index ranges	-23 ≤ h ≤ 21, -24 ≤ k ≤ 24, -17 ≤ l ≤ 39
θ range (°)	3.396 – 62.498
Measured reflections and unique reflections	112742 / 39941 (R_{int} =0.0375)
Goodness-of-fiton F^2	0.992
Largest diff. peak/hole / e $Å^{-3}$	5.2/-3.4
Final R indexes [I>=2σ (I)]	R_1 = 0.0443, wR_2 = 0.1184
Final R indexes [all data]	R_1 = 0.0577, wR_2 = 0.1133

Table S4. Crystal data and structure refinement for the $[Ag_{34}(S\text{-}Adm)_{18}(DPPP)_3Cl_4](SbF_6)_2$ nanocluster. The CCDC number of $[Ag_{34}(S\text{-}Adm)_{18}(DPPP)_3Cl_4](SbF_6)_2$ is 2094273.

Crystal system	trigonal
Space group	R -3
a/Å	26.59(2)
b/Å	26.59(2)
c/Å	87.85(3)
α/°	90
β/°	90
γ/°	120
Volume/$Å^3$	53775(90)
Z	6
ρcalcg/cm^3	1.533
μ/mm^{-1}	16.901
F(000)	24324
Radiation	CuKα (λ = 1.54186)
Index ranges	-30 ≤ h ≤ 24, -21 ≤ k ≤ 30, -37 ≤ l ≤ 101
θ range (°)	6.84 – 58.84
Measured reflections and unique reflections	40279 / 18671 (R_{int} =0.0486)
Goodness-of-fiton F^2	1.281
Largest diff. peak/hole / e $Å^{-3}$	3.7/-1.6
Final R indexes [I>=2σ (I)]	R_1 = 0.1066, wR_2 = 0.3549
Final R indexes [all data]	R_1 = 0.1390, wR_2 = 0.3100

Table S5. Crystal data and structure refinement for the $[Ag_{37}(S\text{-}Adm)_{25}Cl_1]^+$ nanocluster. The CCDC number of $[Ag_{37}(S\text{-}Adm)_{25}Cl_1]^+$ is 2094275.

Crystal system	monoclinic
Space group	P 21/c
a/Å	21.2728(12)
b/Å	36.8575(16)
c/Å	41.407(2)
α/°	90
β/°	103.605(4)
γ/°	90
Volume/Å^3	31554(3)
Z	4
ρcalcg/cm^3	1.727
μ/mm^{-1}	19.859
F(000)	16112
Radiation	CuKα (λ = 1.54186)
Index ranges	-24 ≤ h ≤ 24, -41 ≤ k ≤ 42, -47 ≤ l ≤ 26
θ range (°)	3.762 – 62.500
Measured reflections and unique reflections	218541 / 49672 (R_{int} =0.0748)
Goodness-of-fiton F^2	0.991
Largest diff. peak/hole / e Å^{-3}	3.4/-2.3
Final R indexes [I>=2σ (I)]	R_1 = 0.0670, wR_2 = 0.1894
Final R indexes [all data]	R_1 = 0.0833, wR_2 = 0.1781

Table S6. Crystal data and structure refinement for the $[Ag_{52}(S\text{-}Adm)_{28}Cl_4]^{2+}$ nanocluster (prepared in the presence of DPPPE). The CCDC number of the $[Ag_{52}(S\text{-}Adm)_{28}Cl_4]^{2+}$ nanocluster (prepared in the presence of DPPPE) is 2094276.

Crystal system	orthorhombic
Space group	P c c n
a/Å	22.5930(2)
b/Å	38.2448(3)
c/Å	47.1063(2)
α/°	90
β/°	90
γ/°	90
Volume/$Å^3$	40702.9(5)
Z	4
ρcalcg/cm^3	1.703
μ/mm^{-1}	21.384
F(000)	20240
Radiation	CuKα (λ = 1.54186)
Index ranges	-22 ≤ h ≤ 27, -17 ≤ k ≤ 46, -56 ≤ l ≤ 52
θ range (°)	3.982 – 68.788
Measured reflections and unique reflections	123849 / 35549 (R_{int} =0.1507)
Goodness-of-fiton F^2	0.801
Largest diff. peak/hole / e $Å^{-3}$	1.8/-2.3
Final R indexes [I>=2σ (I)]	R_1 = 0.0690, wR_2 = 0.1977
Final R indexes [all data]	R_1 = 0.1631, wR_2 = 0.1591

Table S7. Crystal data and structure refinement for the $[Ag_{25}(S\text{-}Adm)_{18}][Ag_3(S\text{-}Adm)_2(DPPH)_2]$ nanocluster. The CCDC number of $[Ag_{25}(S\text{-}Adm)_{18}][Ag_3(S\text{-}Adm)_2(DPPH)_2]$ is 2094482.

Crystal system	monoclinic
Space group	P *21/c*
a/Å	21.739(2)
b/Å	20.734(2)
c/Å	33.597(4)
α/°	90
β/°	90.70(1)
γ/°	90
Volume/Å^3	15142.3(3)
Z	2
ρcalcg/cm^3	1.596
μ/mm^{-1}	15.957
F(000)	7240
Radiation	CuKα (λ = 1.54186)
Index ranges	-25 ≤ h ≤ 23, -23 ≤ k ≤ 10, -38 ≤ l ≤ 36
θ range (°)	3.982 – 68.788
Measured reflections and unique reflections	93149 / 23937 (R_{int} =0.0461)
Goodness-of-fiton F^2	0.980
Largest diff. peak/hole / e Å^{-3}	1.8/-1.0
Final R indexes [I>=2σ (I)]	R_1 = 0.0494, wR_2 = 0.1359
Final R indexes [all data]	R_1 = 0.0671, wR_2 = 0.1298

Table S8. Comparison of corresponding bond lengths in different $[Ag_{25}(SR)_{18}]^-$ nanoclusters.

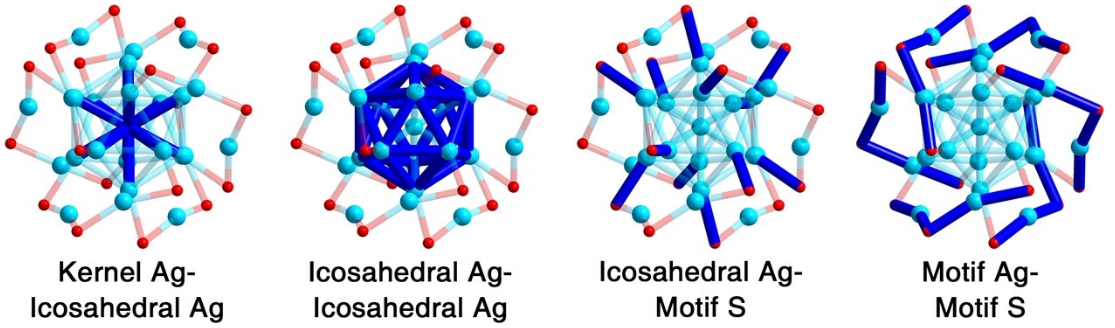

Cluster	Kernel Ag-Icosahedral Ag		Icosahedral Ag-Icosahedral Ag		Icosahedral Ag-Motif S		Motif Ag-Motif S	
	Range (Å)	Avg.(Å)	Range (Å)	Avg.(Å)	Range (Å)	Avg.(Å)	Range (Å)	Avg.(Å)
**Ag_{25}(S-PhMe$_2$)$_{18}$ (Ref 1)**	2.749-2.785	2.764	2.821-2.998	2.907	2.453-2.510	2.479	2.382-3.025	2.500
**Ag_{25}(S-Adm)$_{18}$ (Ag_{25}-DPPE)**	2.753-2.808	2.777	2.870-2.975	2.920	2.450-2.503	2.479	2.380-3.056	2.454
Diff.	-	+0.47%	-	+0.45%	-	+0%	-	-1.84%
**Ag_{25}(S-Adm)$_{18}$ (Ag_{25}-DPPH)**	2.753-2.806	2.773	2.855-2.970	2.916	2.434-2.508	2.476	2.354-3.013	2.475
Diff		+0.33%		+0.31%		-0.12%		-1.00%

Ref 1. $[Ag_{25}(SPhMe_2)_{18}]^-$ referring "C. P. Joshi, M. S. Bootharaju, M. J. Alhilaly, O. M. Bakr. $[Ag_{25}(SR)_{18}]^-$: The "Golden" Silver Nanoparticle. *J. Am. Chem. Soc.*, 2015, **137**, 11578-11581".

Ag_{25}-DPPE. $[Ag_{25}$(S-Adm)$_{18}$][Ag_1(DPPE)$_2$]

Ag_{25}-DPPH. $[Ag_{25}$(S-Adm)$_{18}$][Ag_3(S-Adm)$_2$(DPPH)$_2$]

Table S9. Comparison of Ag-Cl or Ag-S bond lengths in different silver^{-} nanoclusters. Notes: the Ag-S bonds in this table represent the interactions between Ag and the sole S atoms without any carbon tails rather than the interactions between Ag and S-Adm ligands.

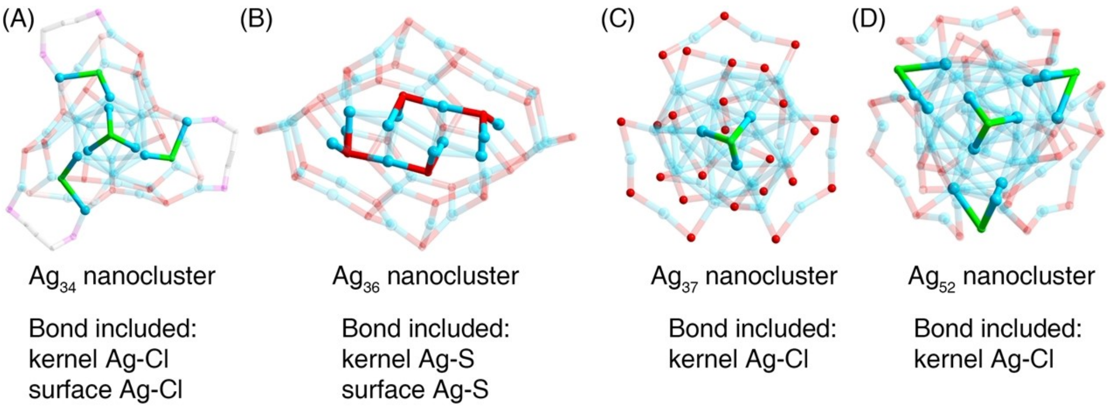

Cluster	kernel Ag-Cl/S		Surface Ag-Cl/S	
	Range (Å)	Avg.(Å)	Range (Å)	Avg.(Å)
**Ag_{34} nanocluster (Ag-Cl)**	2.694-2.694	2.694	2.811-2.811	2.811
**Ag_{36} nanocluster (Ag-S)**	2.379-2.467	2.417	2.623-2.668	2.645
**Ag_{37} nanocluster (Ag-Cl)**	2.753-2.806	2.540	-	-
**Ag_{52} nanocluster (Ag-Cl)**	2.691-2.872	2.778	-	-
